# Parallel functional assessment of m^6^A sites in human endodermal differentiation with base editor screens

**DOI:** 10.1038/s41467-022-28106-0

**Published:** 2022-01-25

**Authors:** Weisheng Cheng, Fang Liu, Zhijun Ren, Wenfang Chen, Yaxin Chen, Tianwei Liu, Yixin Ma, Nan Cao, Jinkai Wang

**Affiliations:** 1 Department of Medical Informatics, Zhongshan School of Medicine, Sun Yat-sen University, 510080 Guangzhou, China; 2 Center for Stem Cell Biology and Tissue Engineering, Key Laboratory for Stem Cells and Tissue Engineering, Ministry of Education, Sun Yat-sen University, 510080 Guangzhou, China; 3 Department of Clinical Laboratory, the First Affiliated Hospital of Anhui Medical University, 230022 Hefei, China; 4 Department of Genetics and Cell Biology, Zhongshan School of Medicine, Sun Yat-sen University, 510080 Guangzhou, China; 5 RNA Biomedical Institute, Sun Yat-sen Memorial Hospital, Sun Yat-sen University, 510120 Guangzhou, China

**Keywords:** RNA modification, Stem-cell differentiation, Functional genomics, Epigenetics

## Abstract

*N*^6^-methyladenosine (m^6^A) plays important role in lineage specifications of embryonic stem cells. However, it is still difficult to systematically dissect the specific m^6^A sites that are essential for early lineage differentiation. Here, we develop an adenine base editor-based strategy to systematically identify functional m^6^A sites that control lineage decisions of human embryonic stem cells. We design 7999 sgRNAs targeting 6048 m^6^A sites to screen for m^6^A sites that act as either boosters or barriers to definitive endoderm specification of human embryonic stem cells. We identify 78 sgRNAs enriched in the non-definitive endoderm cells and 137 sgRNAs enriched in the definitive endoderm cells. We successfully validate two definitive endoderm promoting m^6^A sites on *SOX2* and *SDHAF1* as well as a definitive endoderm inhibiting m^6^A site on *ADM*. Our study provides a functional screening of m^6^A sites and paves the way for functional studies of m^6^A at individual m^6^A site level.

## Abstract (web summary)

N6-methyladenosine (m^6^A) plays important role in lineage specifications of embryonic stem cells, but its role at specific sites has not been assessed. Here the authors develop an adenine editor-based strategy, and systematically identify functional m^6^A sites that control lineage decisions in human embryonic stem cells.

## Introduction

In mammal cells, *N*^6^-methyladenosine (m^6^A) is the most abundant internal chemical modification in messenger RNA and non-coding RNA, transcriptomic identification of m^6^A sites has revealed their strong enrichment in the DRA^*^CH (A^*^ denotes *N*^6^-methylated adenosine) motif in the last exons^1,2^. m^6^A modification is installed co-transcriptionally by the METTL3-METTL14-WTAP core methyltransferase complex and erased by the demethylases ALKBH5 and FTO mainly in the nucleus^1,3,4^. A number of RNA-binding proteins, especially the YTH domain-containing proteins, can specifically bind to m^6^A loci as the m^6^A ‘readers’ and mediate a variety of downstream post-transcriptional effects, including RNA decay, translation, RNA structure switch, and nuclear export^5^. So far, m^6^A has been reported to be involved in a variety of physiological and pathological processes^6,7^.

We and others previously found that depletion of the m^6^A methyltransferase complex results in blocked differentiation in both human and mouse embryonic stem cells (hESCs and mESCs)^8,9^, illuminating that m^6^A methylation, which serves as a timely maintainer of the balance between pluripotency and lineage priming factors, is crucial in regulating cellular specification during embryogenesis. These pioneer studies have shown that m^6^A in mRNA may work as a ‘plug-in’ to other pre-existing pathways by altering downstream gene expression. In this manner, m^6^A modifications can promote fast responses to external cues during times of cell fate transition, thus inspiring studies at the emerging tunable layer termed epitranscriptome. However, such studies are limited by the bulk nature of these experiments in which the methylation level of thousands of sites is altered. To date, it is still difficult to systematically dissect the specific m^6^A sites that are essential for early lineage differentiation due to the lack of a high-throughput screening method for functional m^6^A modification.

**Fig. 1:**
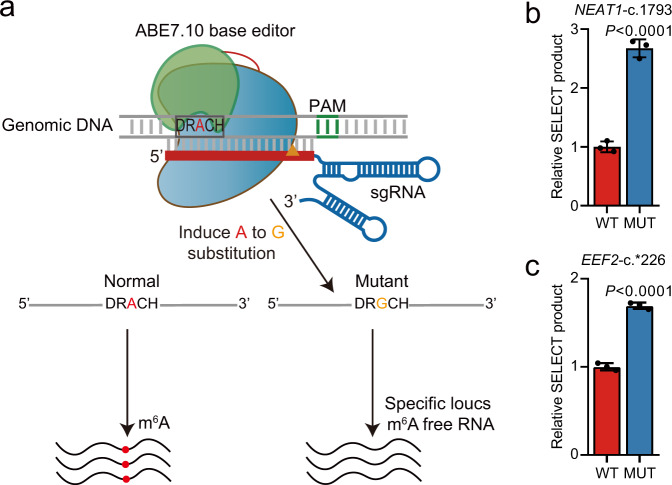
Adenine base editor can induce mutation at m^6^A site. **a** Schematic representation of adenine base editor-mediated mutation at m^6^A site. **b**, **c** Comparisons of m^6^A levels between A549 wild-type (WT) cells and mutant (MUT) cells with FNLS-ABE7.10(AW) induced *NEAT1*-c.1793 (**b**) and *EEF2*-c.*226 (**c**) mutations at m^6^A sites using SELECT assay (*n* = 3 biologically independent samples). Data are presented as means ± SD. *P*-values were calculated vs. WT (two-tailed Student’s *t*-test). Source data are provided as a Source Data file.

CRISPR/Cas9 genome editing that induces double-strand DNA breaks (DSBs) has been widely used for genome-wide screening of essential genes in a variety of biological assays^10^. By fusing Cas9n (Cas9^D10A^), which is a Cas9 mutant that causes single-strand nick, with a cellular deaminase, two types of Cas9-based DNA base editors have been recently developed^11,12^. The cytosine base editors (CBEs) use the rat cytidine deaminase enzymes APOBEC1 (apolipoprotein B mRNA editing enzyme, catalytic polypeptide-like 1) to convert cytidine to uridine on DNA, while the adenine base editors (ABEs) use an evolved *Escherichia coli* tRNA adenosine deaminase (ecTadA) to convert adenine to inosine, which is treated as guanine by polymerases^11,12^. CBEs and ABEs can achieve cytosine to thymine and adenine to guanine substitution, respectively, with low indel frequency and high editing efficiency. Very recently, three groups reported successful large-scale functional screening of genetic variants or mutations using CBEs^13–15^. Because editing an m^6^A site to guanine on the genome theoretically disrupts the corresponding m^6^A modification on the RNA transcribed, it is possible that ABEs targeting the m^6^A sites can be developed for functional m^6^A screening in a high-throughput and transcriptome-wide manner (Fig. 1a).

In this study, we developed an adenine base editor-based strategy to systematically identify functional m^6^A sites that control lineage decisions of hESCs at a transcriptome-wide scale. We designed 7999 sgRNAs targeting 6048 m^6^A sites to screen for m^6^A sites that may act as either boosters or barriers to definitive endoderm (DE) specification of hESCs using a marker of DE CXCR4 (chemokine (C-X-C motif) receptor 4)^16^. We found that 78 sgRNAs were enriched in the CXCR4^−^ non-DE cells and 137 sgRNAs were enriched in the CXCR4^+^ DE cells. We validated two identified DE-promoting m^6^A sites *SOX2* and *SDHAF1* as well as a DE-inhibiting m^6^A site on *ADM* can affect DE specification via promoting the RNA decay of the corresponding genes. Our study provides a functional screening of m^6^A sites at a transcriptome-wide scale and paved the way for studying the functions of m^6^A modification at the individual m^6^A site level.

## Results

### ABE can sufficiently disrupt m^6^A modification

First of all, we tested whether base editing was technically feasible for functional screening. Lentivirus-mediated stable transfection, which usually results in much lower transgene expression when compared to the liposome-based transient transfection^17^, is, unfortunately, a prerequisite in a recessive genetic screen. Thus, we used the codon-optimized Cas9n (RA-Cas9n)-derived base editors, which can remarkably increase the translation efficiency of Cas9 and increase the editing efficiency by about 15-fold^18^. We first modified the lentiviral expression vector of BE3 and ABE7.10(AW) to generate FNLS-BE3 and FNLS-ABE7.10(AW) by substituting Cas9 sequence with an extensively optimized coding sequence of BE3^18^ or low RNA off-targeting mutant ABE7.10 base editor ABE7.10(AW)^19^, followed by adding Flag tag and nuclear localization signal (NLS) at N-terminus (FNLS). We successfully introduced them into A549 cells (a lung carcinoma cell line) with high expression efficiency under continuous antibiotics selection (Supplementary Fig. 1a–h). We found that FNLS-BE3 and FNLS-ABE7.10(AW) nearly completely substituted their targeted nucleotides on *HEK4* and *METTL3*-1 locus, respectively (Supplementary Fig. 1d, h). We further successfully edited two high-confidence m^6^A sites on *NEAT1* and *EEF2* gene, which were detected by both m^6^A-CLIP-seq^1^ and miCLIP-seq^20^ in multiple cell types, using FNLS-ABE7.10(AW) (Supplementary Fig. 2a–d) and confirmed that the m^6^A methylation at these sites was significantly reduced using the SELECT method^21^ (Fig. 1b, c). Since lentivirus may get silenced in embryonic stem cells^22,23^, we analyzed the Cas9 expression of both FNLS-BE3 and FNLS-ABE7.10(AW) in the established hESC cell line with lentiviral transduction. Unfortunately, the expression of FNLS-BE3 was silenced as early as five passages. However, we found robust expression of FNLS-ABE7.10(AW) remained virtually unchanged after continuous culture for 3 months (20 passages) with high homogeneous (>95%) (Supplementary Fig. 3), providing a reliable system that was not compromised by transgene silencing.

### BE-based functional screening exhibits sufficient power

**Fig. 2:**
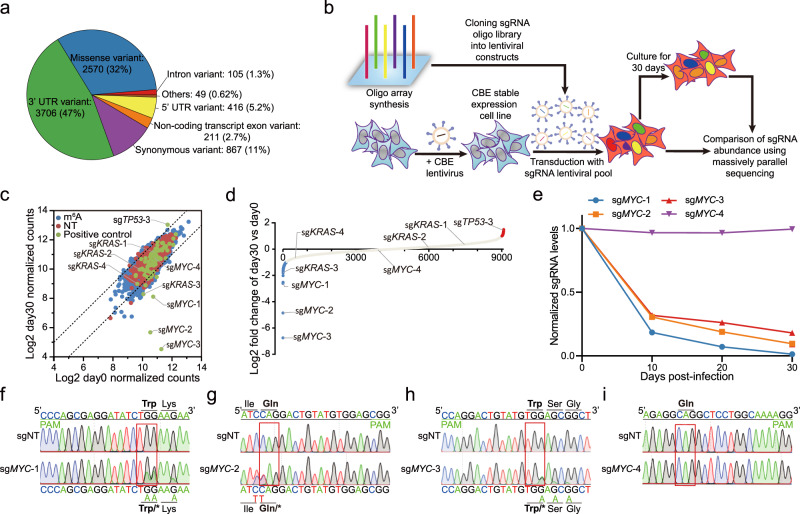
FNLS-BE3 base editor-based screening in A549 cells. **a** Variant effect prediction of FNLS-ABE7.10(AW) base editor-induced mutations for targetable loci. **b** Schematic illustration depicting FNLS-BE3 base editor-based screening in A549 cells. **c** Scatter plot comparing the normalized sgRNA counts of day 0 and day 30 during FNLS-BE3 base editor-based screening in A549 cells. The dotted lines indicate log2-fold change (LFC) of 1 and −1, respectively. **d** Dot plot showing the sorted LFCs of sgRNAs in FNLS-BE3 base editor-based screening in A549 cells. sgRNAs with LFC < −1 or LFC > 1 were colored in blue or red, respectively. **e** Relative abundances of *MYC* targeting sgRNAs predicted to induce BE3-mediated CRISPR-STOP during the expansion of A549 cells (*n* = 2 biologically independent samples). Data are presented as means. **f**–**i** Representative sequence chromatogram of FNLS-BE3-treated A549 cells at *MYC*-1 (**f**), *MYC*-2 (**g**), *MYC*-3 (**h**), and *MYC*-4 (**i**) loci. Source data are provided as a Source Data file.

We were then curious about whether the modified base editors could be used for high-throughput screening of functional m^6^A loci. We designed 7999 sgRNAs targeting 6048 m^6^A sites identified by m^6^A-CLIP-seq^1^ as well as 1000 non-targeting sgRNAs from Human GeCKO v2 library as the negative control (Supplementary Data 1). Based on Variant effect prediction (VEP) tools^24^, we found that 47% of these m^6^A sites locate at 3′UTR of the targeted genes, whereas 32% of them cause potential missense mutation by ABE base editor (Fig. 2a). Because the design space for the sgRNAs of base editors is extremely restricted, 74.2% of these m^6^A sites are targeted by single sgRNAs, implying that it is difficult to maximize the sensitivities and confidence that can be achieved by multiple sgRNAs.

To clarify whether these base editors exhibit sufficient power to enrich functional sgRNAs, we first tested whether CBE-caused premature termination codons^25^, which would more dramatically affect the functions of targeted genes, could be captured in a functional screening. Therefore, we designed an additional 77 sgRNAs that cause premature termination codons by FNLS-BE3 on oncogenes, such as *MYC* and *KRAS*, as well as tumor suppressor genes such as *TP53*. We then combined these sgRNAs with the m^6^A-targeting sgRNA library and the non-targeting controls to screen for sgRNAs that affect the proliferation of A549 cells using FNLS-BE3 (Fig. 2b).

After continuous cultivation for 30 days, 3 sgRNAs causing premature termination of *MYC* (sg*MYC*-1,2,3) and 1 sgRNA of *KRAS* (sg*KRAS*-3) were significantly depleted during long-term expansion, while sgRNA that caused premature termination of *TP53* was significantly enriched in the remaining cells (Fig. 2c–e, Supplementary Fig. 4a, b, and Supplementary Data 2). Sanger sequencing revealed that all these significant sgRNAs of *TP53*, *MYC*, and *KRAS* had high editing efficiencies (Fig. 2f–h, and Supplementary Fig. 4c, d). To test whether the other non-significant sgRNAs were due to inefficient base editing, we also measured the editing efficiencies for the non-significant sgRNA of *MYC* (sg*MYC*-4) and *KRAS* (*sgKRAS*-1,2,4) (Fig. 2e, and Supplementary Fig. 4b). As expected, these non-significant sgRNAs cannot edit the m^6^A sites at all (Fig. 2i, and Supplementary Fig. 4e–g), suggesting that editing efficiencies of the BE sgRNAs are important factors for the outcomes of screening. Taken together, those results indicated that the base editing systems are promising for functional screening.

### Screening of critical m^6^A sites for human DE specification

**Fig. 3:**
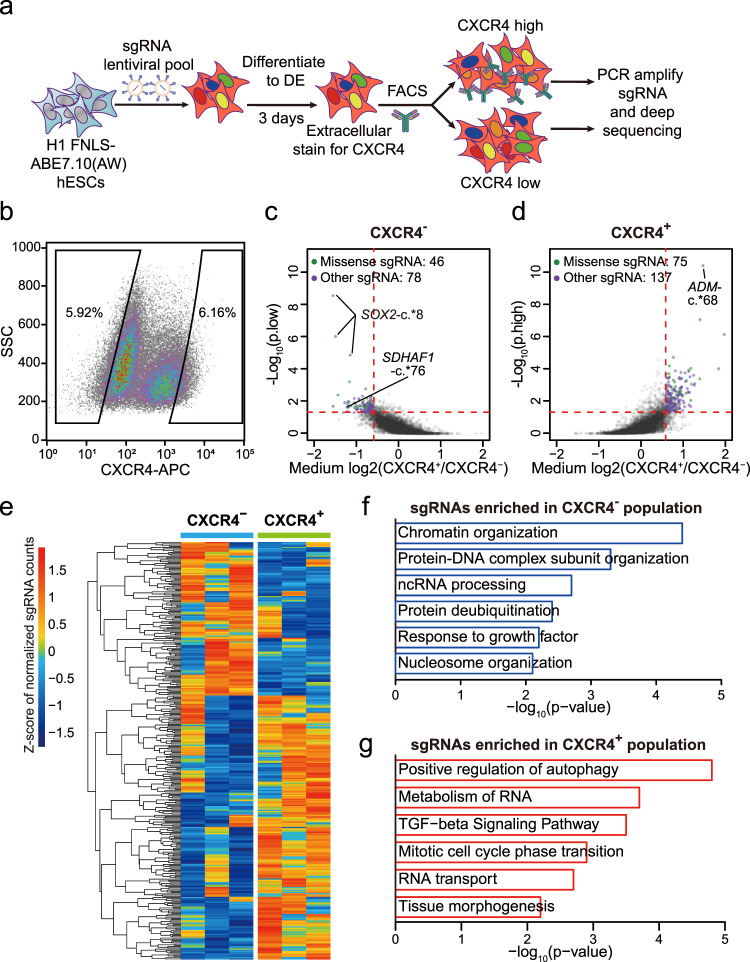
FNLS-ABE7.10(AW) base editor-based functional screening of m^6^A sites in H1 hESCs. **a** Flow chart of ABE-based screening of m^6^A sites is important for endoderm specification. **b** Representative CXCR4 FACS gating strategy for sorting CXCR4^−^ and CXCR4^+^ populations, respectively. SSC side scatter. **c**, **d** Scatter plot showing the strategy to determine the sgRNAs significantly enriched in CXCR4^−^ (**c**) and CXCR4^+^ (**d**) populations, respectively. **e** Heatmap showing the normalized counts of sgRNAs enriched in CXCR4^−^ or CXCR4^+^ populations for each replicate. **f**, **g** GO-enrichment analyses of the genes targeted by the sgRNAs significantly enriched in the CXCR4^−^ (**f**) or CXCR4^+^ (**g**) populations, respectively.

Next, we utilized the screening platform established above and performed high-throughput genetic screens to interrogate questions regarding the site-specific effects of m^6^A in early differentiation events during human embryonic development. We used FNLS-ABE7.10(AW) to screen for m^6^A sites that may act as either boosters or barriers to DE specification of hESCs in three biological replicates. We firstly constructed a stable ABE base editor hESC line by infecting the H1 hESCs with FNLS-ABE7.10(AW) virus followed by antibiotic selection for the infected cells. Then, we transduced the ABE-containing hESCs with a lentiviral library of m^6^A-targeting sgRNAs, induced DE differentiation with a relative inefficient differentiation protocol (~60%) according to previous study^26^ to facilitate the subsequent isolation of both CXCR4^+^ DE and CXCR4^−^ non-DE cells by fluorescence-activated cell sorting (FACS) (Fig. 3a, b, and Supplementary Fig. 5). The abundance of individual sgRNAs in each population was determined by Illumina sequencing. The sgRNA sequencing counts resemble normal distributions in both undifferentiated hESCs and after DE differentiation (Supplementary Fig. 6a, b). We found the sgRNAs strongly overrepresented in CXCR4^+^ (LFC > 1) had significantly higher editing efficiencies (Supplementary Fig. 6c). This is consistent with our finding that editing efficiency is important for the outcome of CBE-based screening. Furthermore, the sgRNAs strongly overrepresented in CXCR4^−^ (LFC < –1) had significantly higher gene expression of H1 hESCs^8^, suggesting that these sgRNAs were enriched mainly through the effects on their targeted RNAs rather than off-target effects or direct interaction with the DNA (Supplementary Fig. 6d).

To determine the individual functional m^6^A sites in endodermal differentiation, we calculated the *P* values using MAGeCK software^27^ based on the three replicates of independent screens. Similar to the previous base editor screening of functional nucleotide variants^13^, we required *P* < 0.05 and absolute fold change >1.5 (LFC > 0.58) to determine the significantly enriched sgRNAs in CXCR4^−^ and CXCR4^+^ populations, respectively (Fig. 3c, d). According to these criteria, 1.9% and 1.7% of the 1000 non-targeting sgRNAs were significantly enriched in CXCR4^−^ and CXCR4^+^ populations, respectively, suggesting a relatively low false-positive rate (Supplementary Data 3). Although the distributions of different types of sgRNAs are not significantly altered in the significantly enriched sgRNAs in CXCR4^−^ or CXCR4^+^ populations, the significant sgRNAs predicted to induce missense mutations had a trend of enrichment in both CXCR4^−^ or CXCR4^+^ populations (from 32% to 37% and 35%, respectively) (Supplementary Fig. 6e–g), suggesting that a small subset of sgRNAs may get enriched through changing the amino acids other than disrupting m^6^A. We, therefore, filtered out the sgRNAs predicted to induce missense mutations for downstream analyses. We finally identified 75 m^6^A sites targeted by 78 sgRNAs were significantly enriched in the CXCR4^−^ population (Fig. 3c), while 137 m^6^A sites targeted by 137 sgRNAs were significantly enriched in the CXCR4^+^ population (Fig. 3d). As shown in Fig. 3e, these significant sgRNAs are highly reproducible across the three independent replicates. The genes targeted by CXCR4^−^ population enriched sgRNAs are enriched in pluripotency-related gene ontology (GO) terms such as “chromatin organization” and “nucleosome organization”. Whereas the genes targeted by sgRNAs enriched in CXCR4^+^ population are enriched in GO term that promotes stem cell differentiation, such as “TGF-beta signaling pathway” and “tissue morphogenesis”, which is consistent with the notion that degradation of these RNAs through m^6^A will inhibit the differentiation (Fig. 3f, g).

If using more stringent criteria by requiring absolute LFC > 1 and *P* < 0.05, none of the significant sgRNAs in CXCR4^+^ population came from non-targeting sgRNAs (Supplementary Fig. 6h, i), indicating a low false discovery rate based on these criteria. We then used these criteria to determine 12 high-confidence m^6^A sites targeted by 14 sgRNAs enriched in the CXCR4^−^ population as well as 19 high-confidence m^6^A loci targeted by 19 sgRNAs enriched in the CXCR4^+^ population (Supplementary Data 3). We, therefore, refer to the m^6^A sites targeted by these significant sgRNAs using stringent criteria as high-confidence m^6^A sites. We found 24 out of them (75%) target the m^6^A sites located in 3′UTRs, which is the region the m^6^A mostly likely to occur. Three sgRNAs targeting common m^6^A sites of *SOX2*, which is a known master regulator of hESCs that leads to impaired DE differentiation when overexpressed^28^, turned out to be the sgRNAs with the most significant *P* values enriched in CXCR4^−^ populations (Fig. 3c), indicating the screening is effective.

In addition, we compared the normalized sgRNA counts in hESCs before DE induction with CXCR4^−^ and CXCR4^+^ cells. For sgRNAs significantly enriched in CXCR4^+^ and CXCR4^−^, respectively, we found that the normalized sgRNA counts in hESCs were overall in the middle of CXCR4^−^ and CXCR4^+^ cells, suggesting most of the CXCR4^+^ and CXCR4^−^ enriched sgRNAs are due to their effects on DE specification (Supplementary Fig. 6j, k). On the other hand, we also observed 119 sgRNAs with normalized counts in hESCs more than 2-fold higher than both CXCR4^+^ and CXCR4^−^ populations, suggesting they may be toxic to hESCs (Supplementary Fig. 6l). Consistently, these sgRNAs are significantly enriched in genes related to apoptosis and regulation of cell growth (Supplementary Fig. 6m). Whereas there were also 62 sgRNAs with normalized counts in hESCs more than 2-fold lower than both CXCR4^+^ and CXCR4^−^ populations, suggesting that they may confer proliferative advantages during the DE specification (Supplementary Fig. 6l, m; Supplementary Data 3). Consistently, these sgRNAs are significantly enriched in genes related to GO terms related to proliferation, including “positive regulation of cell growth” and “response to insulin” (Supplementary Fig. 6m).

### Selected m^6^A disruptive mutations increase RNA stabilities

We performed validation experiments for the high-confidence m^6^A sites that also exhibit the highest degree of methylation in hESCs revealed by m^6^A-LAIC-seq^29^, including non-DE-enriched hits *SOX2*-c.*8 and *SDHAF1*-c.*76, as well as DE-enriched hit *ADM*-c.*68. Besides *SOX2*, the roles of the other two genes in DE differentiation have not been reported. *SDHAF1* encodes succinate dehydrogenase (SDH) complex assembly factor 1 that is essential for SD assembly in the mitochondria^30,31^; *ADM*, which encodes adrenomedullin, is a multifunctional regulatory peptide consisting of 52 amino acids and synthesized by a large number of tissues and cells^32^. We further confirmed that all of the three loci were highly m^6^A modified in hESCs by m^6^A-seq (Supplementary Fig. 7a–c).

**Fig. 4:**
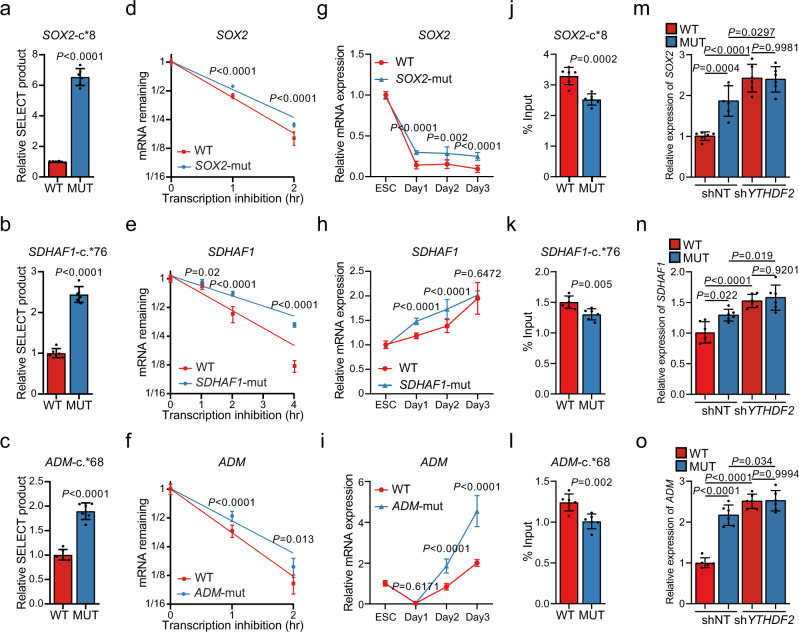
Selected m^6^A disruptive mutations increase RNA stabilities depending on *YTHDF2*. **a**–**c** Comparisons of SELECT-determined m^6^A levels at the targeted sites in *SOX2*-mut (**a**), *SDHAF1*-mut (**b**), and *ADM*-mut (**c**) hESCs with WT hESCs (*n* = 6 biologically independent samples). *P*-values were calculated vs. WT (two-tailed Student’s *t*-test). **d**–**f** mRNA half-life (*t*_1/2_) of *SOX2* (**d**), *SDHAF1* (**e**), or *ADM* (**f**) in WT, and mutant hESCs (*n* = 6 biologically independent samples). *P*-values were calculated vs. WT (two-tailed Student’s *t*-test). **g**–**i** mRNA expression levels of *SOX2* (**g**), *SDHAF1* (**h**), and *ADM* (**i**) in cultures derived from WT, *SOX2*-mut, *SDHAF1*-mut, and *ADM*-mut hESCs before and after DE differentiation measured by qPCR (*n* = 8 biologically independent samples in (**g**), *n* = 6 biologically independent samples in (**h**) and (**i**)). *P*-values were calculated vs. WT (two-tailed Student’s *t*-test). **j**–**l** RIP-qPCR analyses of the YTHDF2 binding at the mRNAs of *SOX2* (**j**), *SDHAF1* (**k**), and *ADM* (**l**) in cultures derived from WT and mutant hESCs at day 2 of DE differentiation (*n* = 6 biologically independent samples). *P*-values were calculated vs. WT (two-tailed Student’s *t*-test). **m**–**o** mRNA expression levels of *SOX2* (**m**), *SDHAF1* (**n**), and *ADM* (**o**) in cultures derived from WT and mutant (MUT) hESCs at day 2 of DE differentiation with or without *YTHDF2* knockdown (*n* = 6 biologically independent samples). NT non-targeting; ns nonsignificant (one-way ANOVA with Tukey’s post hoc test). Data of all relevant panels are presented as means ± SD. Source data are provided as a Source Data file.

Based on a non-integrated base editing strategy, we generated clonal *SOX2*-c.*8A>G (*SOX2*-mut), *SDHAF1*-c.*76A>G (*SDHAF1*-mut), and *ADM*-c.*68A>G (*ADM*-mut) homozygosis mutant (mut) hESCs, respectively (Supplementary Fig. 7d–f). During passaging, all of them retained a stable growth rate, undifferentiated morphology, high alkaline phosphatase activity (Supplementary Fig. 8a), and uniform expression of key pluripotent marker NANOG, OCT4, and SOX2 (>99%) (Supplementary Fig. 8b), as well as the proliferation marker Ki67 (Supplementary Fig. 8c). Meanwhile, they were completely stained negative for the three germ-layer genes (Supplementary Fig. 9a–d). These results suggest that the mutant hESCs retain an undifferentiated state before endodermal induction, consistent with the previous reports that m^6^A of ES cells is not necessary for self-renewal and growth^8^. To test whether m^6^A modification was erased at the mutant sites, we performed SELECT analyses and observed significant increases of ligated products in all of the three mutants, suggesting evident decreases of m^6^A deposition at the targeted sites (Fig. 4a–c).

Since the major role of m^6^A plays in cell fate transition is promoting the mRNA degradation^8,33^, we examined the abundance and turnover rate of *SOX2*, *SDHAF1*, and *ADM* mRNA in WT control and the mutant hESCs. Notably, we observed substantial increases of half-lives (Fig. 4d–f) in the mutant, suggesting that site-specific m^6^A modification is sufficient to regulate mRNA decay. Upon induction of DE specification, we found significantly up-regulated expression of *SOX2, SDHAF*1, and *ADM* in the mutant cells (Fig. 4g–i), consistent with the known role of m^6^A in ES cells that primes the transcripts for degradation upon signaling of differentiation^34^. To test whether these effects were mediated by YTHDF2, the major m^6^A reader that facilitates RNA decay through reading m^6^A^33^, we performed YTHDF2 RIP-qPCR and confirmed the binding between YTHDF2 and *SOX2*, *SDHAF1*, or *ADM* mRNA at day 2 of DE differentiation, which was decreased with the *SOX2*-c.*8A>G, *SDHAF1*-c.*76A>G, or *ADM*-c.*68A>G mutation (Fig. 4j–l). Notably, we found the up-regulation of mRNA abundance of *SOX2*, *SDHAF1*, and *ADM* on day 2 of DE differentiation by the mutations at their corresponding m^6^A sites were completely abolished by *YTHDF2* knockdown (Fig. 4m–o, and Supplementary Fig. 10a). These results suggest that these m^6^A sites affect DE specification by regulating the RNA stabilities in a YTHDF2-dependent manner.

### Selected m^6^A mutations regulate human DE specification

**Fig. 5:**
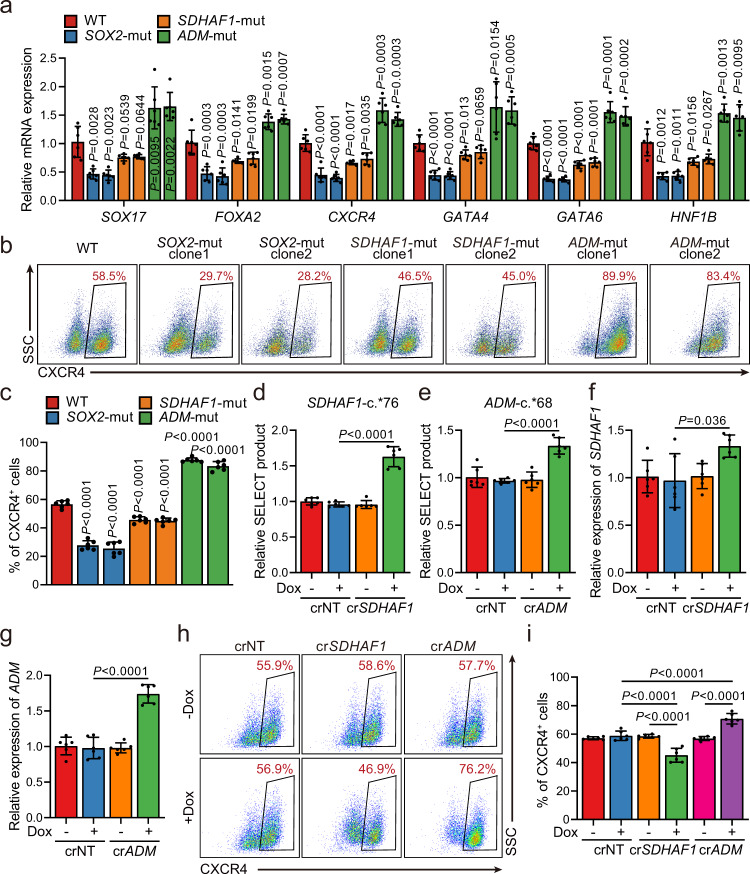
Selected m^6^A-disruptive mutations regulate human endodermal specification through m^6^A. **a** mRNA levels of DE marker genes *SOX17*, *FOXA2*, *CXCR4*, *GATA4*, *GATA6*, and *HNF1B* in cultures derived from WT, *SOX2*-mut, *SDHAF1*-mut, and *ADM*-mut as well as WT hESCs at day 3 of DE differentiation (*n* = 6 biologically independent samples). *P*-values were calculated vs. WT (two-tailed Student’s *t*-test). **b**, **c** Representative (**b**) and quantitative (**c**) flow cytometry analyses of CXCR4 expression in cultures derived from WT, *SOX2*-mut, *SDHAF1*-mut, and *ADM*-mut hESCs at day 3 of DE differentiation (*n* = 6 biologically independent samples). *P*-values were calculated vs. WT (one-way ANOVA with Tukey’s post hoc test). **d**, **e** Measurement of m^6^A levels of *SDHAF1*-c.*76 (**d**) and *ADM*-c.*68 (**e**) after TRME editor induced site-specific m^6^A demethylation by SELECT assay (*n* = 6 biologically independent samples). NT non-targeting, Dox doxycycline (one-way ANOVA with Tukey’s post hoc test). **f**, **g** mRNA expression levels of *SDHAF1* (**f**) and *ADM* (**g**) on day 2 of DE differentiation after TRME editor induced site-specific m^6^A demethylation (*n* = 6 biologically independent samples). NT non-targeting, Dox doxycycline (one-way ANOVA with Tukey’s post hoc test). **h**, **i** Representative (**h**) and quantitative (**i**) flow cytometry analyses of CXCR4 expression in cultures derived from hESCs with or without induced site-specific m^6^A demethylation by TRME editor at day 3 of DE differentiation (*n* = 6 biologically independent samples). NT non-targeting, Dox doxycycline (one-way ANOVA with Tukey’s post hoc test). Data of all relevant panels are presented as means ± SD. Source data are provided as a Source Data file.

We then determined whether site-specific modulation of the above m^6^A sites identified in the screening would affect the DE specification of hESCs. Upon induction of differentiation, the differentiated populations are a mixture that contains both undifferentiated hESCs and their endodermal derivatives, with different efficiency dependents on the mutation that the cells carried (Supplementary Fig. 10b–e). Based on the mean fluorescence intensity (MFI) of SOX2, we found a significant increase of the percent of SOX2^+^ cells together with a significant increase of MFI of SOX2 per cell for SOX2^+^ cells (Supplementary Fig. 11a–c). In addition, we observed significant decreases in the expression of many key DE genes in *SOX2-* and *SDHAF1-*mutant hESCs (Fig. 5a–c, and Supplementary Fig. 12a–c), consistent with the fact that these two mutations were found to be enriched in the CXCR4^−^ population in the primary screens. In contrast, *ADM*-c.*68A>G mutation, which was enriched in the CXCR4^+^ DE cells, had an opposite effect and significantly promoted the DE specification of hESCs as expected (Fig. 5a–c, and Supplementary Fig. 12a–c).

To exclude the possibility that the effects of the sgRNA-induced mutations on DE differentiation are through DNA other than m^6^A on RNAs, we investigated the regional effects of m^6^A modification removal at *SDHAF1*-c.*76 and *ADM*-c.*68 without changing the primary genomic DNA sequences. To achieve this goal, we used our previously developed dCas13a-ALKBH5-based doxycycline-inducible targeted RNA m^6^A erasure (TRME) system^35^, by which we have previously demonstrated m^6^A erasure at the *SOX2*-c.*8 site inhibited DE specification of the hESCs. With the presence of doxycycline, we observed significantly decreased m^6^A deposition (Fig. 5d, e) in undifferentiated hESCs as well as significantly increased mRNA levels of *SDHAF1* or *ADM* on day 2 of DE differentiation (Fig. 5f, g) only in dCas13a-ALKBH5 hESCs harboring the *SDHAF1*-c.*76- or *ADM*-c.*68-targeting but not non-targeting (NT) crRNAs. Upon induction of DE specification, we found that doxycycline-treatment in the dCas13a-ALKBH5 hESCs significantly decreased the percentage of CXCR4^+^ or SOX17^+^/FOXA2^+^ endodermal cells with the presence of *SDHAF1*-c.*76 crRNA, whereas cells harboring the *ADM*-c.*68-targeting crRNA generated DE cells more efficiently (Fig. 5h, i, and Supplementary Fig. 13a–c). These data were consistent with the results showing *SDHAF1*-c.*76A>G mutation inhibits but *ADM*-c.*68A>G mutation promotes DE specification, indicating the effects of these mutation causing sgRNAs on DE specification are truly m^6^A based.

### *SDHAF1* and *ADM* are regulators of DE specification

**Fig. 6:**
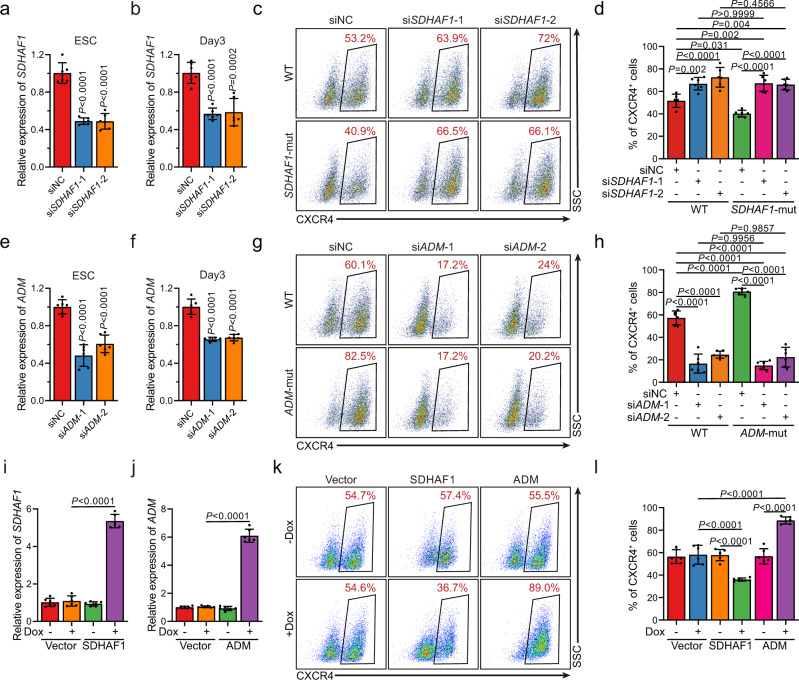
The mRNAs of *SDHAF1* and *ADM* mediate the effects of their targeting sgRNAs on DE specification. **a**, **b** siRNA knockdown efficiency of *SDHAF1* in hESCs (**a**) and at day 3 of DE differentiation (**b**), respectively (*n* = 6 biologically independent samples), measured by RT-qPCR. si siRNA, NC non-targeting control. *P*-values were calculated vs. siNC (one-way ANOVA with Tukey’s post hoc test). **c**, **d** Representative (**c**) and quantitative (**d**) flow cytometry analyses of CXCR4 expression in cultures derived from WT or *SDHAF1-*mut hESCs that with NC or *SDHAF1*-targeting siRNAs at day 3 of DE differentiation (*n* = 6 biologically independent samples). ns non-significant (one-way ANOVA with Tukey’s post hoc test). **e**, **f** siRNA knockdown efficiency of *ADM* in hESCs (**e**) and at day 3 of DE differentiation (**f**) respectively (*n* = 6 biologically independent samples), measured by RT-qPCR. si siRNA, NC non-targeting control. *P*-values were calculated vs. siNC (one-way ANOVA with Tukey’s post hoc test). **g**, **h** Representative (**g**) and quantitative (**h**) flow cytometry analyses of CXCR4 expression in cultures derived from WT or *ADM*-mut hESCs that with NC or *ADM*-targeting siRNAs at day 3 of DE differentiation (*n* = 6 biologically independent samples). ns non-significant (one-way ANOVA with Tukey’s post hoc test). **i**, **j** mRNA levels of *SDHAF1* (**i**) and *ADM* (**j**) in overexpressed hESCs (*n* = 6 biologically independent samples), measured by RT-qPCR. Dox, doxycycline (one-way ANOVA with Tukey’s post hoc test). **k**, **l** Representative (**k**) and quantitative (**l**) flow cytometry analyses of CXCR4 expression in cultures derived from WT and overexpressed hESCs at day 3 of DE differentiation (*n* = 6 biologically independent samples). Dox doxycycline (one-way ANOVA with Tukey’s post hoc test). Data of all relevant panels are presented as means ± SD. Source data are provided as a Source Data file.

Since *SDHAF1* and *ADM* were less studied in hESCs, we further characterized them in DE specification. We found that transient short interfering RNA (siRNA) knockdown of *SDHAF1* expression in hESCs led to improved DE specification (Fig. 6a–d, and Supplementary Fig. 14a, c, d), whereas *ADM* knockdown cells formed DE cells expressing CXCR4, SOX17, and FOXA2 at much lower efficiency (Fig. 6e–h, and Supplementary Fig. 14b, e, f). Consistently, overexpression of *SDHAF1* suppressed the DE specification, whereas overexpression of *ADM* improved DE specification, suggesting that these genes are involved in hESCs-DE transition (Fig. 6i–l, and Supplementary Fig. 15a–c). More importantly, knockdown of *SDHAF1* or *ADM* completely abolished the effects of *SDHAF1*-c.*76A>G and *ADM*-c.*68A>G mutation on DE specification, further validating that the phenotypes induced by m^6^A ablation were arisen from regulating the target genes but not caused by other putative off-targets (Fig. 6c, d, g, h). In aggregate, these results collectively establish that site-specific m^6^A modulation is sufficient to produce distinct lineage choice outcomes in hESCs, further highlighting the importance of m^6^A-dependent epitranscriptional control in cell fate transitions.

## Discussion

Although the functions of m^6^A have been revealed in a variety of physiological and pathological processes, most of these discoveries are based on disruption of the methyltransferases, demethylases, or readers. These proteins are known to target a large number of m^6^A sites, however, due to the lack of functional screening methods, the causal relationships between the presence of a specific m^6^A and the phenotype observed have never been systematically identified. Here, we demonstrate that ABE base editor can be used to functionally access tens of thousands of m^6^A sites in a pooled screening at base resolution. It offers researchers a versatile toolbox to systematically dissect the specific m^6^As underlying the phenotypic outcomes. Application of this system in human endodermal specification successfully uncovered critical m^6^A sites that either boost or inhibit the DE specification of hESCs.

Our screening of functional m^6^A sites provided insights into the epitranscriptomic mechanisms in the human endodermal specification. The results indicated that m^6^A modification on a considerable number of genes within a variety of pathways was required in cell fate transition and disrupting any of these critical sites rather than genetic perturbations of the m^6^A writers, erasers, or readers, is sufficient to change the cell fate. It in turn indicated that transcriptome-wide functional screening of m^6^A was important for elucidating the detailed epitranscriptomic mechanisms of cell fate transition and very possibly many other biological processes regulated by m^6^A (Fig. 3f, g). On the other hand, the modification of those m^6^A sites that play important roles in a common process might be coordinated and co-regulated by upstream specific regulators of m^6^A, such as RNA-binding proteins and transcription factors^34–37^.

As we have proved in this study, editing efficiency is critical for the success of base editing screens. High transfection efficiency is the key factor for high editing efficiency. However, it is known that stable gene transfection, in which a certain number of transgene copies are inserted into the host genome, usually results in lower transgene expression when compared to the liposome-based transient transfection which allows for robust transgene expression from tens of thousands of transgene copies independent of genomic and epigenetic control^17^. In addition, lentivirus-mediated transgene tends to silence during long-term culture, especially in the hESCs^22,23^. We thus used the codon-optimized Cas9n, which remarkably increased the translation efficiency of Cas9 to overcome this disadvantage. Furthermore, though lentivirus-mediated random transgene insertion lacks site-specificity, it generates multiple integration locus within the genome which may reduce the likelihood of the construct being silenced and provides the advantage of a rapid and efficient means of generating stable hESCs transgene clone. In addition, targeting genomic safe harbors, such as the AAVS1 site, may further increase transgene expression levels and homogeneity for future base editor screens especially in ESCs^26^.

The endodermal specification is known to be regulated by a cascade of transcription factors and signal transduction pathways^26,38–40^. Our work not only demonstrated the power of using unbiased an adenine base editor-based strategy to identify the m^6^A sites but also provided previously unreported regulators for DE differentiation: SDHAF1 and ADM. SDHAF1 is essential for the assembly and activity of succinate dehydrogenase, which has been described to be causative for mitochondrial disease^31^. Mutations in *SDHAF1* cause an early-onset onset leukoencephalopathy^31,41^. The defective tissues of the patients are differentiated from endoderm, which indicates SDHAF1 is a booster for ectoderm and its developmental derivatives but not for endoderm. *SDHAF1* knockdown moderately improved the differentiation efficiency of DE further verifying our hypothesis that SDHAF1 is a barrier for endoderm development. ADM is a multifunctional protein that incorporates multiple biological functions associated with the earliest stages of embryo development^42^. A previous study demonstrated that *ADM*^–/–^ is embryonic lethality because of extreme hydrops fetalis and cardiovascular defects^43^. SDHAF1 and ADM have never been reported to be crucial regulators for endoderm development, further suggesting that m^6^A is a different layer of regulation. Although the gene expression changes mediated by post-transcriptional degradation of the m^6^A modified genes can also be achieved by transcriptional regulation mediated by transcription factors, the m^6^A layer may have additional meaning to endoderm development. As we and collaborators have proposed, m^6^A is important for timely cleaning up the RNAs that may maintain the previous cell fate^8^. On the other hand, m^6^A may increase the dynamics of RNAs and make cells more responsive to stimulates. Therefore, functional screening of m^6^A site is important for understanding the different layers of mechanism in cell fate transition.

Although Batista et al. reported that *METTL3* knockdown led to a profound block in endodermal differentiation in multiple H1 hESCs clones^8^, Bertero et al. found that knockdown of the m^6^A methyltransferase complex subunits inhibits neural but not endodermal differentiation of H9 hESCs cell line^34^, which is controversial with Batista et al. This discrepancy may arise from the differences in knockdown efficiencies, a cell line of choice (H1 vs. H9), and hESCs culture conditions (CDM supplemented with FGF2/activin-A vs. mTeSR1) in the two studies. Moreover, Bertero et al. adopted a more complex DE differentiation condition which contains FGF2, LY294002 (a PI3K inhibitor), activin-A, and BMP4, in contrast to the protocol used in Batista et al. in which activin-A is the only DE-inducing reagent. Therefore, components in Bertero et al.’s protocol may bypass the requirement of m^6^A methyltransferase complex subunits in DE specification. For example, PI3K inhibition may down-regulate the expression of some pluripotent genes that serve as a barrier to DE differentiation and thus mimic the effects of m^6^A-mediated degradation of these genes^44^.

It is widely accepted that there are on average only 3–5 m^6^A sites on a single gene, clustering of m^6^A sites was also reported based on single-nucleotide resolution technology^1,20^. It remains elusive whether modification of a single m^6^A site has a considerable functional effect. Our study demonstrates that disruption of single m^6^A sites rather than global m^6^A remodeling can be sufficient to affect stem cells to adopt new cell fates. Since the writers, erasers, and readers of m^6^A usually target too many m^6^A sites, targeting these proteins may have serious side effects in research and clinical treatment, therefore, targeting the specific m^6^A sites using advanced technology may be of great advantage in the future.

Compared with traditional CRISPR/Cas9-based screening of functional genes, ABE-based screening of functional m^6^A sites present unique challenges. By designing multiple sgRNAs that target the same gene, traditional gene screening has superior statistical sensitivities and specificities of the outcomes. However, because the design spaces for the sgRNAs of ABE are extremely restricted, only a single sgRNA can be designed for most of the m^6^A sites. In addition, the sgRNA of ABE has to compromise on the requirements of high efficiencies, low off-target possibilities, implying inevitable sacrifices of sensitivities and accuracies. Furthermore, a single sgRNA of ABE can edit multiple nucleotides in the same editing window, which may induce phenotypes that are not regulated by that m^6^A site. Therefore, as reported in recent CBE-based screenings^13,14^, experimental validation is a critical step for BE-based screenings. On the other hand, mutations of the m^6^A sites on DNA may affect the phenotype through non-m^6^A mechanisms. For example, a substantial proportion of m^6^A targeting sgRNAs also cause missense mutations. Therefore, further experimental validations using Cas13-based epitranscriptional editing on RNAs and uncovering the mechanisms underlying the phenotype are also important for elucidating the genuine causal m^6^A sites.

With the rapid development of CRISPR-based technology, some recent advances provide a promising prospect of reversible regulation of m^6^A modifications. By fusing nuclease-inactive DNA-targeting Cas9 (dead Cas9, dCas9) with ALKBH5, FTO, or METTL3-METTL14, Qian and co-workers developed the m^6^A editing tools by combination with an antisense oligonucleotide (PAMer) to supply the protospacer adjacent motif (PAM), the tools can achieve site-specific demethylation or methylation of RNAs^45^. However, the PAMer oligo synthesized with mixed DNA and 2′OMe RNA bases in vitro requires transient transfection to enter the cells and has a very short half-life, thus is not suitable for high throughput screening of functional m^6^A loci. Meanwhile, Cas13 fused with methyltransferase or demethylase can directly install or remove m^6^A modification on specific sites on RNAs^46–48^, it might be a much better system for functional screening of m^6^A if the editing efficiency and specificity of these editors are significantly improved in the future. Furthermore, a powerful and accurate statistic algorithm that can identify the genuine enrichment of sgRNAs for base editing is also in urgent need.

Although additional work lies ahead to further optimize the efficiency, to broaden the frame of targetable m^6^A loci, and to explore the underlying mechanisms of how the identified m^6^A sites affect lineage specification, the present study represents a key step toward unlocking the secrets of cell fate control at the epitranscriptome layer. Given the broad applicability of the strategy and the versatility of base editor toolkits on the rise, our approach described here may be developed to allow scalable functional characterization of m^6^A modification in many other biological and disease models for similar purposes.

## Methods

### Cell culture

HEK293T (ATCC^®^ CRL-3216™) and A549 (ATCC^®^ CCL-185™) cells were cultured in high glucose Dulbecco’s modified Eagle’s medium (Hyclone, SH30022.01), supplemented with 10% fetal bovine serum (FBS, Hyclone, SH30406.05) and 2 mM GlutaMAX (Gibco, 35050061) at 37 °C with 5% CO_2_. H1 hESCs (WiCell Research Institute) and the TRME hESCs cell line^35^, were grown in Matrigel (BD, 354277)-coated six-well plates in E8 medium (Stem Cells Technology, 05940) at 37 °C with 5% CO_2_. Both cells were authenticated and tested for the absence of mycoplasma contamination using Myco-Blue Mycoplasma Detector (Vazyme).

### DE differentiation of hESCs

Differentiation of hESCs into DE cells was adopted from the previous study with minor modifications^26^. In brief, undifferentiated hESCs were dissociated into single-cell suspension by Accutase (Gibco, A1110501) and reseeded onto Matrigel-coated 24-well plates at a density of 1 × 10^5^ cells/per well in E8 medium containing 10 μM Y-27632 (Selleck, S1049). When reached 80% confluency, DE differentiation was initiated by switching to the differentiation medium DMEM/F-12 (Gibco, 11330032) supplemented with 50 U ml^−1^ Penicillin–Streptomycin (Gibco, 15070063), chemically defined lipid concentrate (1:100, Gibco, 11905031), 10.7 μg ml^−1^ holo-Transferrin human (Sigma, T0665), 71 μg ml^−1^ l-ascorbic acid (Sigma, A8960), 14 ng ml^−1^ sodium selenite (Sigma, S5261), and 20 ng ml^−1^ Activin A (PeproTech, 12014E) and cultured for 3 days. CHIR99021 (3 μM, Selleck, S2924) was added to the medium for the first 24 h of differentiation and removed thereafter.

### Construction of plasmid DNA

Lenti-FNLS-BE3-P2A-Puro-U6-sgRNA was generated using the ClonExpress II One Step Cloning Kit (Vazyme, C112), by combining the PCR-amplified FNLS-BE3 segment from pLenti-FNLS-P2A-Puro (Addgene, #110841) and the *Age* I/*Bam*H I-digested LentiCRISPR v2 (Addgene, #52961) backbone. Lenti-FNLS-ABE7.10(AW)-P2A-Puro-U6-sgRNA was generated using the ClonExpress MultiS One Step Cloning Kit (Vazyme, C113) by combining the synthesized ecTadA(E59A)-ecTadA*(V106W) fragment, PCR-amplified codon-optimized Cas9n segment from Lenti-FNLS-P2A-Puro (Addgene, #110841), and the *Age* I/*Bam*H I digested LentiCRISPR v2 (Addgene, #52961) backbone. U6-sgRNA fragment free FNLS-ABE7.10(AW) expression plasmid (Lenti-FNLS-ABE7.10(AW)-P2A-Puro) was generated by ligating the fragment of *Kpn* I/*EcoR* I-digested Lenti-FNLS-ABE7.10(AW)-P2A-Puro-U6-sgRNA using T4 DNA Ligase (New England Biolabs, M0202). LentiGuide-BSD-dTomato was generated by combining the PCR-amplified blasticidin S deaminase segment from pgRNA-CKB (Addgene, #73501) and the *Eco*R I/*Xba* I-digested LentiGuide-Hygro-dTomato (Addgene, #99376) backbone.

For sg*HEK4*, sg*MYC*-1−4, sg*KRAS*-1−4, and sg*TP53*-3, Lenti-FNLS-BE3-P2A-Puro-U6-sgRNA was used as a backbone, and Lenti-FNLS-ABE7.10(AW)-P2A-Puro-U6-sgRNA was used for sg*METTL3*-1, sg*EEF2*-2, sg*NEAT1*, sg*SOX2*, sg*ADM*, and sg*SDHAF1*. For sg*YTHDF2*, pLKO.1-blast was used as a backbone. All sgRNA/shRNA-inserted plasmids were constructed following the standard protocol of Target Guide Sequence Cloning Protocol from Dr. Feng Zhang’s laboratory (Havard University).

For the TRME assay, crRNAs with target m^6^A site at the 3rd base were designed. Then, full-length DR together with the U6 promoter was PCR-amplified from the pC016-LwCas13a plasmid backbone (Addgene, #91906) and cloned into the pSLQ1371 vector using the ClonExpress II One Step Cloning Kit (Vazyme, C112).

For *SDHAF1* and *ADM* overexpression, coding sequence (CDS) of *SDHAF1* and *ADM* were PCR amplified from open reading frames (ORF) plasmid purchase from Vigenebio (China), and cloned into the pLVX-TetOne-puro vector using the ClonExpress II One Step Cloning Kit.

sgRNAs, shRNAs, or crRNAs sequences used in this study were provided in Supplementary Table 1.

### Lentiviral production and transduction

Lentivirus was packaged by co-transfection of HEK293T cells with 12 μg of lentiviral vector, 3 μg of pMD2.G (Addgene #12259), and 9 μg of psPAX2 (Addgene #12260) using Lipofectamine 2000 reagent (Invitrogen, 11668019). Lentivirus-containing media was harvested and filtered with a 0.45 µm PVDF filter (Millipore). Cells were transduced with the virus in the presence of 8 μg ml^−1^ polybrene. 48 h later, infected cells were selected with 1 µg ml^−1^ puromycin (Selleck, S7417) or 10 μg ml^−1^ blasticidin (Selleck, S7419).

### Design and construction of the sgRNA library

The flanking sequence (30 nucleotides upstream and 30 nucleotides downstream of m^6^A-CLIP-seq^1^ identified single-nucleotide m^6^A sites) was extracted from the genome sequence according to the coordinate (GRCh37) of m^6^A loci for targetable analysis. Then, for each m^6^A site, we searched all possible sgRNAs with m^6^A sites in the editing window by sliding the editing window for every single nucleotide. To construct the sgRNA library, pooled oligonucleotides containing coding sequences of sgRNA and adapter were synthesized and cloned into the LentiGuide-BSD-dTomato vector by GENEWIZ, Inc. Lentiviral particles of the sgRNA library were produced, concentrated, and titered by GENEWIZ, Inc.

### Base-editing screening

For FNLS-BE3 screening, A549 FNLS-BE3 cells were generated by transduced with the pLenti-FNLS-P2A-Puro lentivirus, and infected cells were selected by 1 μg ml^−1^ puromycin for 5 days. Then A549 FNLS-BE3 cells were infected by the sgRNA library lentiviral particles with a low MOI of 0.3 with the presence of 8 μg ml^−1^ of polybrene. 48 h after transduction, infected cells were selected by 10 μg ml^−1^ blasticidin for 5 days. Then, 5 × 10^6^ cells were collected to measure the frequency of each sgRNA in the initial pool (referred to as day 0). The rest of the cells were continually cultured and passaged. 5 × 10^6^ cells were collected on days 10, 20, and 30, respectively.

For FNLS-ABE7.10(AW) screening, H1 FNLS-ABE7.10(AW) hESCs were generated by transduced with the pLenti-FNLS-ABE7.10(AW)-P2A-Puro lentivirus and infected cells were selected by 1 μg ml^−1^ puromycin for 5 days. Then the whole population of H1 FNLS-ABE7.10(AW) hESCs was infected with the sgRNA library lentiviral particles with a low MOI of 0.3 with the presence of 8 μg ml^−1^ of polybrene. 48 h after transduction, infected cells were selected by 10 μg ml^−1^ blasticidin. After 5 days of selection, 5 × 10^6^ cells were collected to measure the frequency of each sgRNA in the initial pool (referred to as day 0), and 5 × 10^6^ cells were reseeded onto two Matrigel-coated 24-well plates for DE differentiation. 3 days later, differentiated DE cultures were stained with an APC-conjugated anti-CXCR4 antibody (Invitrogen, 17-9999-42) and ~6% cells with the lowest or highest CXCR4 expression were collected by FACS, respectively, according to the relative number of transduced sgRNAs vs. the number of cells (3–4 × 10^6^ cells per sample). Three independent FNLS-ABE7.10(AW) screenings were performed from sgRNA library virus infection to FACS, and six sorted cell samples together with the one sample before DE induction were sequenced separately for subsequent data analyses.

### High-throughput sequencing

Genomic DNA (gDNA) was extracted using the FastPure Cell/Tissue DNA Isolation Mini Kit (Vazyme, DC102) and DNA concentration was measured by Qubit using the Qubit™ dsDNA HS Assay Kit (Invitrogen). To generate sgRNA amplicons, we used a single-step PCR protocol which was adopted from the protocol published^49^. All the gDNA harvested from the screenings was used for PCR amplification in 50 µl PCR reactions. Each reaction consisted of 2.5 µg gDNA plus water, 25 μl NEBNext Ultra II Q5 PCR Master Mix, 1.25 μl 10 μM stagger forward primer, and 1.25 μl 10 μM barcoded reverse primer. PCR reactions were cycling as follow: initial denature 3 min at 98 °C; followed by 30 s denature at 98 °C, 10 s anneal at 63 °C, 25 s extension at 72 °C, for 23 cycles; and final extension for 2 min at 72 °C. PCR products were size-selected using VAHTS DNA Clean Beads (Vazyme) according to the manufacturer’s instructions and sequenced on a HiSeq2000 sequencer (Illumina).

### High-throughput sequencing data analyses

Raw single-end reads were trimmed using Cutadapt to remove the constant flanking sequences of sgRNA sequences. Read counts of the sgRNAs were measured using the count command of MAGeCK^27^, the read count of each sgRNA was then normalized by the total reads mapped to all sgRNAs.

For BE3-based screening, log2-fold change (LFC) of normalized counts between day 30 and day 0 samples were calculated. The sgRNAs with absolute LFC > 1 were determined as significantly upregulated or downregulated sgRNAs.

For ABE-based screening, test command of MAGeCK^27^ was used to calculate the raw *P* values for the comparison between CXCR4^−^ versus CXCR4^+^ sgRNA. 33 sgRNAs with three replicates averaged MAGeCK normalized read counts of CXCR4^−^ or CXCR4^+^ samples <200 were removed for further analyses. The medium LFC (log2 (CXCR4^+^/CXCR4^−^)) of three replicates was used in the downstream analyses, which was calculated as the medium of the LFC of three individual replicates. sgRNA with p.high < 0.05 and medium LFC > 0.58 (fold change > 1.5) were used to determine the significantly enriched sgRNA in CXCR4^+^ population. sgRNA with p.low < 0.05 and medium LFC < −0.58 were used to determine the significantly enriched sgRNA in CXCR4^−^ population. The locations and consequences of the sgRNA-induced mutations were predicted by VEP (Ensembl Variant Effect Predictor)^24^ with CDS has the highest priority across all isoforms, followed by 3'-UTR, 5'-UTR, intron, intergenic. sgRNAs predicted to induce missense mutations were filtered out in the identification of significantly enriched sgRNAs. Metascape^50^ was used to perform GO analysis for the genes targeted by the significant sgRNAs. Editing efficiencies of the sgRNAs were predicted by a machine learning algorithm BE-Hive^51^. FPKM (Fragments Per Kilobase of transcript per Million mapped reads) of H1 hESCs were directly obtained from the previous publication^8^. Heatmap was plotted using R package pheatmap. We used absolute medium LFC > 1 to identify the differential sgRNAs when comparing the hESCs with CXCR4^+^ or CXCR4^−^ population.

The m^6^A peaks and gene expression of H1 hESCs were obtained directly from our previous publication^8^. The single-nucleotide m^6^A sites of m^6^A-CLIP-seq^1^ and miCLIP-seq^20^ were also obtained directly from the previous publications. The m^6^A peaks of A549 cells were identified using the published m^6^A-seq data^52^ with the method described in our previous publication^8^.

### Flow cytometry and cell sorting

In brief, differentiated DE cultures were rinsed with DMEM/F-12 and dissociated with Accutase for 10–15 min at 37 °C. Cells were washed twice with ice-cold wash buffer (2% FBS in Dulbecco’s PBS, DPBS), resuspended in ice-cold blocking buffer (5% FBS in DPBS), and then incubated with the primary antibody CXCR4-APC for 1 h at 4 °C. Then cells were washed three times, resuspended with ice-cold wash buffer, and examined by a CytoFLEX S Flow Cytometer (Beckman Coulter) or sorted by the FACS MoFlo Astrios EQs system (Beckman Coulter). Data were analyzed by the FlowJo Software (FlowJo LCC). Cells incubated with the APC-conjugated isotype (Invitrogen, 17-4724-81) served as negative controls.

### Genomic DNA extraction, PCR amplification, and Sanger sequencing

Genomic DNA was isolated using FastPure Cell/Tissue DNA Isolation Mini Kit (Vazyme, DC102), and genomic regions of interest were amplified by using a 2×Phanta Max Master Mix (Vazyme, P511) according to the manufacturer’s instructions. Purified DNA was sequenced by an ABI 3730xl DNA Analyzer (Applied Biosystems) and analyzed using SnapGene (GSL Biotech LLC). Primer sequences used for target amplification were provided in Supplementary Table 2.

### Plasmid and siRNA transfection

To introduce point mutations into hESCs, 1 × 10^6^ H1 hESCs were transfected with 2 μg base editor plasmid using Lipofectamine Stem transfection reagent (USA, Invitrogen, stem00008) following the manufacturer’s instructions. 24 hours after transfection, transfected cells were selected with 1 µg ml^−1^ puromycin for 48 h and reseeded onto Matrigel-coated 6 cm dishes at 5 × 10^3^ cells per dish with the presence of CloneR (StemCell, 05888) following the manufacturer’s instructions. Single cell-derived clones were picked about 7 days later, amplified in culture, and then genotyped by Sanger sequencing of the gRNA-targeted site. For siRNA knockdown experiments, 1 × 10^5^ H1 hESCs were transfected with the siRNA oligo (50 nM final siRNA concentrations) using DharmaFECT1 transfection reagents (Dharmacon, T-2001) following the manufacturer’s instructions. Knockdown efficiencies of siRNA-targeted genes were detected by real-time quantitative PCR (RT-qPCR). siRNA sequences used in this study were provided in Supplementary Table 3.

### TRME cell line construction

NKX2-5eGFP/w hESCs were dissociated into single-cell suspension by Accutase and reseeded onto Matrigel-coated 24-well plates at a density of 1 × 10^5^ cells/per well in E8 medium containing 10 μM Y-27632 and cells were co-nucleofected with TRME editor plasmid (dCas13a-ALKnes)^35^ and transposase plasmid at a mass ratio of 1000:1 using the Neon^®^ Transfection System (Thermo Fisher Scientific). 24 h after transfection, cells were treated 1 μg ml^−1^ doxycycline with daily media change until stable colonies appeared. Then, cells were transduced with crRNA-expressing lentiviruses, and cells with both GFP and mCherry expression were sorted by FACS and expanded for further experiments.

### RNA binding protein immunoprecipitation (RIP)

EZ-Magna RIP™ RNA-Binding Protein Immunoprecipitation Kit (Sigma-Aldrich, 17-701) following the manufacturer’s instructions. The anti-YTHDF2 (Proteintech, 24744-1-AP, 5 μg per sample) antibody was used for RIP. The input and IP RNA of each sample was purified and evaluated through RT-qPCR.

### Western blot

Cells were lysed in RIPA buffer (Cell Signaling Technology, 9806) supplemented with 1 mM PMSF and Proteinase inhibitor (Roche, 4693132001). 30 μg protein per lane was fractionated on 6–12% SDS–PAGE and transferred to the PVDF membrane (GE Healthcare Life Sciences). Membranes were blocked in the blocking buffer (DPBS, supplemented with 5% skimmed milk, 0.1% Tween 20) for 1 h at RT. Membranes were then incubated with the primary antibodies including anti-Cas9 (1:3000, Diagenode, C15310258), and anti-β-actin (1:1000, 4A Biotech, 4ab080291) in the antibody dilution buffer (Solarbio) overnight at 4 °C. Then membranes were washed by DPBS containing 0.1% Tween-20, incubated with HRP-conjugated secondary antibody (Beyotime Biotechnology, 1:1000) in antibody dilution buffer for 1 h at RT, and visualized by the Clarity™ Western ECL Substrate (Bio-Rad).

### Alkaline Phosphatase (AP) staining

AP stainings were performed by using the Alkaline Phosphatase Detection Kit (Sigma-Aldrich, SCR004) following the manufacturer’s instructions.

### Immunofluorescence

For immunofluorescence, cells were fixed in 4% paraformaldehyde (Solarbio) for 30 min at RT and washed with 0.3 M glycine in DPBS. Cells were then blocked and permeabilized in the permeabilization buffer (DPBS supplemented with 8% donkey serum, 8% goat serum, and 0.3% Triton X-100) at RT for 1 h. Cells were then stained with the primary antibody in primary antibody statin buffer (DPBS supplemented with 1% BSA, 1% goat serum, and 0.25% Triton-X) at 4 °C overnight. After washing with DPBS containing 0.1% BSA and 0.1% Triton X-100, cells were stained with fluorescent secondary antibody in secondary antibody buffer (DPBS supplemented with 0.05% Triton X-100 and 1% BSA) at RT for 1 h and analyzed using the Operetta CLS system (Perkin Elmer) in the same settings. Primary antibodies included SOX17 (1:200, R&D, AF1924), FOXA2 (1:200, Cell Signaling Technology, 8186S), SOX2 (1:200, Abcam, ab79351), NANOG (1:200, Cell Signaling Technology, 3580S), OCT4 (1:200, Santa Cruz, sc-5279), SOX1 (1:200, Cell Signaling Technology, 4194S), Brachury (1:100, R&D, AF2085), Ki67 (1:100, BD Biosciences, 550609). Secondary antibodies used were Alexa488-conjugated donkey anti-rabbit (1:800, Invitrogen, A21206), Alexa488-conjugated goat anti-mouse (1:800, Invitrogen, A21121), Alexa647-conjugated goat anti-mouse (1:800, Invitrogen, A21242), Alexa555-conjugated goat anti-mouse (1:800, Invitrogen, A21127), Alexa594-conjugated donkey anti-goat (1:800, Invitrogen, A11058).

### SELECT for detection of m^6^A

1 μg total RNA from the control group or expression level normalized RNA from the experimental group was mixed with 40 nM up primer, 40 nM down primer, 5 μM dNTP, 1.7 μl 10× CutSmart buffer (New England Biolabs, B7204), DEPC H_2_O to the final volume 17 μl. The following temperature annealed the mixture of RNA and primers: 1 min at 90 °C, 1 min at 80 °C, 1 min at 70 °C, 1 min at 60 °C, 1 min at 50 °C and 6 min at 40 °C. Subsequently, a 3 μl of enzyme mixture containing 0.01 U *Bst* 2.0 DNA polymerase (New England Biolabs, M0537), 0.5 U SplintR ligase (New England Biolabs, M0375), and 10 nmol ATP was added in the annealing products. The final mixture was incubated at 40 °C for 20 min, heat inactivation at 80 °C for 20 min and stored at 4 °C. qPCR was then performed in QuantStudio^TM^ 7 Flex Real-Time PCR System (Applied Biosystems) using ChamQ Universal SYBR qPCR Master Mix (Vazyme, Q711). Relative SELECT products between the experimental group and control group were calculated using the 2^−∆∆Ct^ method. Primers used in the SELECT assays were provided in Supplementary Table 4.

### RNA stability assay and RT-qPCR

For RNA stability assays, cells were treated with transcription inhibitor actinomycin D (Sigma, A9415) at 5 μg ml^−1^. RNA samples were collected at various time points and analyzed by RT-qPCR. For RT-qPCR, total RNA was extracted using the FastPure Cell/Tissue Total RNA Isolation Kit (Vazyme, RC101). 1 μg of DNA-free total RNA was then reverse-transcribed using HiScript II Q RT SuperMix for qPCR (Vazyme, R223). RT-qPCR was carried out using the ChamQ Universal SYBR qPCR Master Mix (Vazyme, Q711) and performed in a QuantStudio^TM^ 7 Flex Real-Time PCR System (Applied Biosystems). *18S* and *GAPDH* were used as the reference gene in RNA stability assay and gene expression assay, respectively. Relative fold-change was calculated using the 2^−∆∆Ct^ method. The RT-qPCR primer sequences used in this study were provided in Supplementary Table 5.

### Statistics and reproducibility

Graphs and statistical analyses were carried out using GraphPad Prism 8.0 (GraphPad Software Inc.). Statistical significance of differences was estimated by two-tailed unpaired Student’s *t*-test for two groups comparisons and one-way ANOVA with Tukey’s post hoc test for multiple groups comparisons. A *P* value of <0.05 was determined as statistically significant. Data were presented as means ± standard deviation (SD) quantified from at least three biological repeats unless otherwise stated. The immunoblot (Supplementary Fig. 1b, f) and immunostaining (Supplementary Figs. 1c, g, 3, 8b, c, 9b–d) experiments were performed at least three independent times with similar results.

### Reporting summary

Further information on research design is available in the Nature Research Reporting Summary linked to this article.

## Supplementary information

Supplementary Information

Reporting Summary

Description of Additional Supplementary Files

Supplementary Dataset 1

Supplementary Dataset 2

Supplementary Dataset 3

## Data Availability

Source data are provided with this paper. Informations of the designed sgRNA library are provided in the Supplementary Data 1. The sgRNA counts data generated in this study are provided in the Supplementary Data 2 and  3. The raw data generated in this study have been deposited in the GEO database under accession number GSE179980. The original un-cropped images of western blots in this study are provided in the Supplementary Fig. 16. Source data are provided with this paper.

## Source data

Source Data

## Code availability

Source code and analysis scripts for sgRNA design and analyses are available on GitHub (https://github.com/ZJRen9/CRISPR_screen_effective_m6ASite, 10.5281/zenodo.5588306).

## References

[1] Ke SD (2015). A majority of m(6)A residues are in the last exons, allowing the potential for 3 ' UTR regulation. Genes Dev.

[2] Dominissini D (2012). Topology of the human and mouse m(6)A RNA methylomes revealed by m(6)A-seq. Nature.

[3] Jia GF (2011). N6-Methyladenosine in nuclear RNA is a major substrate of the obesity-associated FTO. Nat. Chem. Biol..

[4] Liu JZ (2014). A METTL3-METTL14 complex mediates mammalian nuclear RNA N-6-adenosine methylation. Nat. Chem. Biol..

[5] Zaccara S, Ries RJ, Jaffrey SR (2019). Reading, writing and erasing mRNA methylation. Nat. Rev. Mol. Cell Biol..

[6] Frye M, Harada BT, Behm M, He C (2018). RNA modifications modulate gene expression during development. Science.

[7] Lan Q (2019). The critical role of RNA m(6)A methylation in cancer. Cancer Res..

[8] Batista PJ (2014). m(6)A RNA modification controls cell fate transition in mammalian embryonic stem cells. Cell Stem Cell.

[9] Geula S (2015). m6A mRNA methylation facilitates resolution of naive pluripotency toward differentiation. Science.

[10] Komor AC, Badran AH, Liu DR (2017). CRISPR-based technologies for the manipulation of eukaryotic genomes. Cell.

[11] Komor AC, Kim YB, Packer MS, Zuris JA, Liu DR (2016). Programmable editing of a target base in genomic DNA without double-stranded DNA cleavage. Nature.

[12] Gaudelli NM (2017). Programmable base editing of A.T to G.C in genomic DNA without DNA cleavage. Nature.

[13] Cuella-Martin R (2021). Functional interrogation of DNA damage response variants with base editing screens. Cell.

[14] Hanna RE (2021). Massively parallel assessment of human variants with base editor screens. Cell.

[15] Xu P (2021). Genome-wide interrogation of gene functions through base editor screens empowered by barcoded sgRNAs. Nat. Biotechnol.

[16] D’Amour KA (2006). Production of pancreatic hormone-expressing endocrine cells from human embryonic stem cells. Nat. Biotechnol..

[17] Felgner PL (1987). Lipofection: a highly efficient, lipid-mediated DNA-transfection procedure. Proc. Natl. Acad. Sci. USA.

[18] Zafra MP (2018). Optimized base editors enable efficient editing in cells, organoids and mice. Nat. Biotechnol..

[19] Rees HA, Wilson C, Doman JL, Liu DR (2019). Analysis and minimization of cellular RNA editing by DNA adenine base editors. Sci. Adv.

[20] Linder B (2015). Single-nucleotide-resolution mapping of m6A and m6Am throughout the transcriptome. Nat. Methods.

[21] Xiao Y (2018). An elongation- and ligation-based qPCR amplification method for the radiolabeling-free detection of locus-specific N-6-methyladenosine modification. Angew. Chem.-Int. Ed..

[22] Herbst F (2012). Extensive methylation of promoter sequences silences lentiviral transgene expression during stem cell differentiation in vivo. Mol. Ther..

[23] Xia X, Zhang Y, Zieth CR, Zhang SC (2007). Transgenes delivered by lentiviral vector are suppressed in human embryonic stem cells in a promoter-dependent manner. Stem Cells Dev..

[24] McLaren W (2016). The ensembl variant effect predictor. Genome Biol..

[25] Billon P (2017). CRISPR-mediated base editing enables efficient disruption of eukaryotic genes through induction of STOP codons. Mol. Cell.

[26] Li QV (2019). Genome-scale screens identify JNK-JUN signaling as a barrier for pluripotency exit and endoderm differentiation. Nat. Genet..

[27] Li W (2014). MAGeCK enables robust identification of essential genes from genome-scale CRISPR/Cas9 knockout screens. Genome Biol..

[28] Wang Z, Oron E, Nelson B, Razis S, Ivanova N (2012). Distinct lineage specification roles for NANOG, OCT4, and SOX2 in human embryonic stem cells. Cell Stem Cell.

[29] Molinie B (2016). m(6)A-LAIC-seq reveals the census and complexity of the m(6)A epitranscriptome. Nat. Methods.

[30] Na U (2014). The LYR factors SDHAF1 and SDHAF3 mediate maturation of the iron-sulfur subunit of succinate dehydrogenase. Cell Metab..

[31] Ghezzi D (2009). SDHAF1, encoding a LYR complex-II specific assembly factor, is mutated in SDH-defective infantile leukoencephalopathy. Nat. Genet..

[32] Kitamura K (2012). Adrenomedullin: a novel hypotensive peptide isolated from human pheochromocytoma. Biochem. Biophys. Res. Commun..

[33] Wang X (2014). N-6-methyladenosine-dependent regulation of messenger RNA stability. Nature.

[34] Bertero A (2018). The SMAD2/3 interactome reveals that TGFbeta controls m(6)A mRNA methylation in pluripotency. Nature.

[35] Chen XN (2021). Targeted RNA N-6-methyladenosine demethylation controls cell fate transition in human pluripotent stem cells. Adv. Sci..

[36] Barbieri I (2017). Promoter-bound METTL3 maintains myeloid leukaemia by m(6)A-dependent translation control. Nature.

[37] An S (2020). Integrative network analysis identifies cell-specific trans regulators of m6A. Nucleic Acids Res..

[38] Teo AK (2011). Pluripotency factors regulate definitive endoderm specification through eomesodermin. Genes Dev..

[39] Shi ZD (2017). Genome editing in hPSCs reveals GATA6 haploinsufficiency and a genetic interaction with GATA4 in human pancreatic development. Cell Stem Cell.

[40] Robertson EJ (2014). Dose-dependent Nodal/Smad signals pattern the early mouse embryo. Semin. Cell Dev. Biol..

[41] Ohlenbusch A (2012). Leukoencephalopathy with accumulated succinate is indicative of SDHAF1 related complex II deficiency. Orphanet J. Rare Dis..

[42] Li M, Yee D, Magnuson TR, Smithies O, Caron KM (2006). Reduced maternal expression of adrenomedullin disrupts fertility, placentation, and fetal growth in mice. J. Clin. Investig..

[43] Caron KM, Smithies O (2001). Extreme hydrops fetalis and cardiovascular abnormalities in mice lacking a functional Adrenomedullin gene. Proc. Natl. Acad. Sci. USA.

[44] Singh AM (2012). Signaling network crosstalk in human pluripotent cells: a Smad2/3-regulated switch that controls the balance between self-renewal and differentiation. Cell Stem Cell.

[45] Liu XM, Zhou J, Mao Y, Ji Q, Qian SB (2019). Programmable RNA N(6)-methyladenosine editing by CRISPR-Cas9 conjugates. Nat. Chem. Biol..

[46] Wilson C, Chen PJ, Miao Z, Liu DR (2020). Programmable m(6)A modification of cellular RNAs with a Cas13-directed methyltransferase. Nat. Biotechnol..

[47] Li JX (2020). Targeted mRNA demethylation using an engineered dCas13b-ALKBH5 fusion protein. Nucleic Acids Res..

[48] Xia Z (2021). Epitranscriptomic editing of the RNA N6-methyladenosine modification by dCasRx conjugated methyltransferase and demethylase. Nucleic Acids Res..

[49] Joung J (2017). Genome-scale CRISPR-Cas9 knockout and transcriptional activation screening. Nat. Protoc..

[50] Zhou YY (2019). Metascape provides a biologist-oriented resource for the analysis of systems-level datasets. Nat. Commun..

[51] Arbab M (2020). Determinants of base editing outcomes from target library analysis and machine learning. Cell.

[52] Lin S, Choe J, Du P, Triboulet R, Gregory RI (2016). The m(6)A methyltransferase METTL3 promotes translation in human cancer cells. Mol. Cell.

