# Supplementary material for: Parallel functional assessment of m6A sites in human endodermal differentiation with base editor screens
Source: Nat Commun. 2022 Jan 25;13:478. doi: 10.1038/s41467-022-28106-0 (PMC8789821; doi:10.1038/s41467-022-28106-0)
Supplement: Supplementary file 1 — Supplementary Information [file 41467_2022_28106_MOESM1_ESM.pdf]

Supplementary Information for

Parallel functional assessment of m^{6}A sites in human endodermal differentiation with base editor screens

Weisheng Cheng[1,2,†], Fang Liu[3,4,†], Zhijun Ren[1,2], Wenfang Chen[1,2], Yaxin Chen[1,2], Tianwei Liu[1,2], Yixin Ma[1,2], Nan Cao[2,4,*], Jinkai Wang[1,2,5,*]

[1] Department of Medical Informatics, Zhongshan School of Medicine, Sun Yat-sen University, Guangzhou 510080, China. [2] Center for Stem Cell Biology and Tissue Engineering, Key Laboratory for Stem Cells and Tissue Engineering, Ministry of Education, Sun Yat-sen University, Guangzhou 510080, China. [3] Department of Clinical Laboratory, the First Affiliated Hospital of Anhui Medical University, Hefei 230022, China. [4] Department of Genetics and Cell Biology, Zhongshan School of Medicine, Sun Yat-sen University, Guangzhou 510080, China. [5] RNA Biomedical Institute, Sun Yat-sen Memorial Hospital, Sun Yat-sen University, Guangzhou 510120, China.

[†] These authors contributed equally: Weisheng Cheng, Fang Liu

[*] Corresponding authors

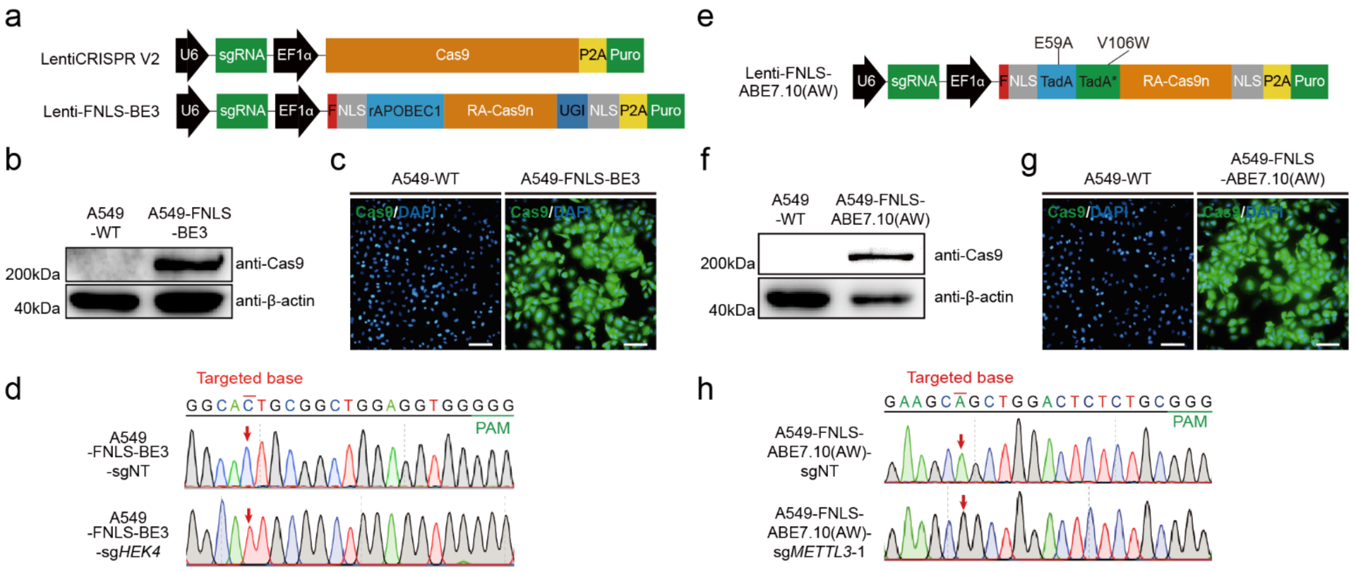

Supplementary Fig. 1 Optimization of the lentiviral expression vector of the base editors. a Schematic diagram of LentiCRISPR v2 and Lenti-FNLS-BE3 constructs. **b** Western blot analyses of FNLS-BE3 expression in A549 cells. β-actin was used as a loading control. **c** Immunofluorescence analyses of FNLS-BE3 expression in A549 cells. Scale bars, 100 μm. **d** Representative sequence chromatogram of FNLS-BE3 treated A549 cells at the *HEK4* locus. Red arrows indicated the conversion of C to T. **e** Schematic representation of Lenti-FNLS-ABE7.10(AW) construct. **f** Western blot analyses of FNLS-ABE7.10(AW) expression in A549 cells. β-actin was used as a loading control. **g** Immunofluorescence analyses of FNLS-ABE7.10(AW) expression in A549 cells. Scale bars, 100 μm. **h** Representative sequence chromatogram of FNLS-ABE7.10(AW) treated A549 cells at the *METTL3*-1 locus. Red arrows indicated the conversion of A to G.

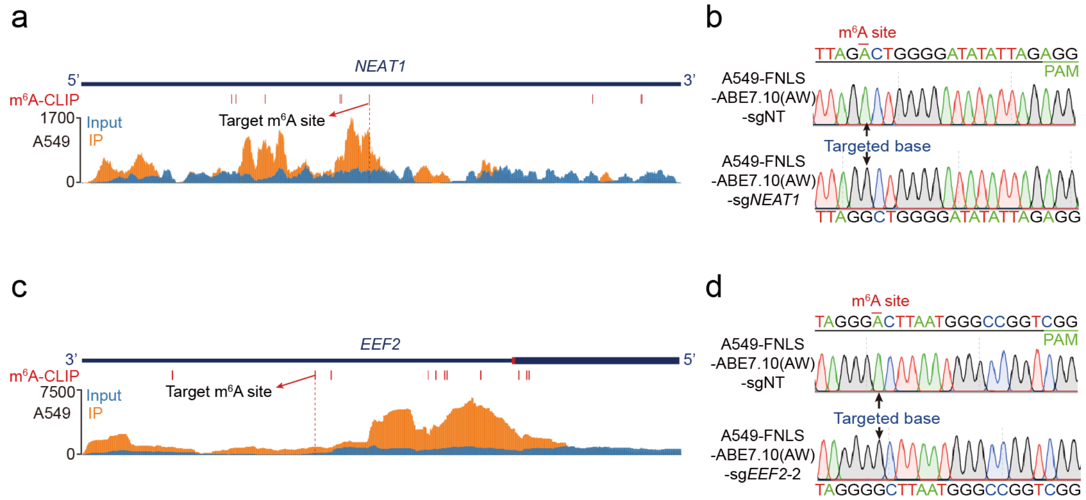

Supplementary Fig. 2 Track demonstration and Sanger sequencing validation of the ABE targetable m^{6}A sites on *NEAT1* and *EEF2* gene in A549 cells. a UCSC genome browser tracks of miCLIP-seq in 293T cells and m^{6}A-CLIP-seq and m^{6}A-seq signals in A549 cells at the *NEAT1* locus. **b** Representative sequence chromatogram of *NEAT1*-mut homozygous mutant A549 monoclonal. **c** UCSC genome browser tracks of miCLIP-seq in 293T cells and m^{6}A-CLIP-seq and m^{6}A-seq signals in A549 cells at the *EEF2* locus. **d** Representative sequence chromatogram of *EEF2*-mut homozygous mutant A549 monoclonal.

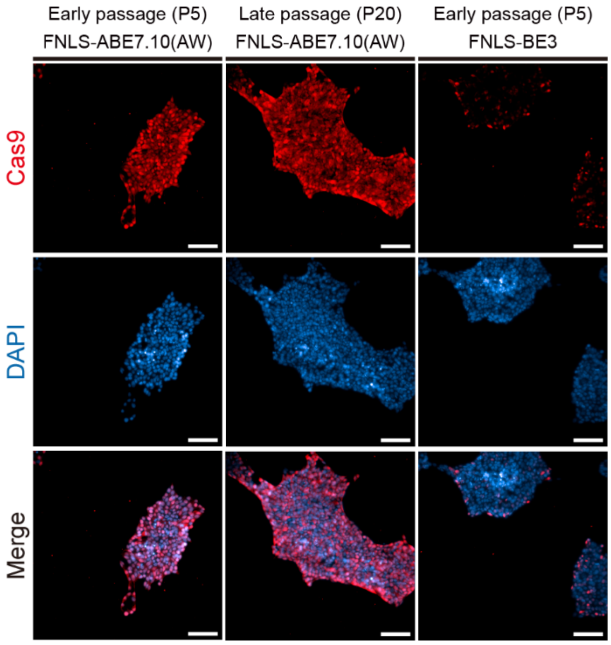

Supplementary Fig. 3 Establishment of base editing system in hESCs. Immunofluorescence analyses of BE3 and ABE base editor expression in H1 hESCs during passaging. P, Passages. Scale bars, 100 µm.

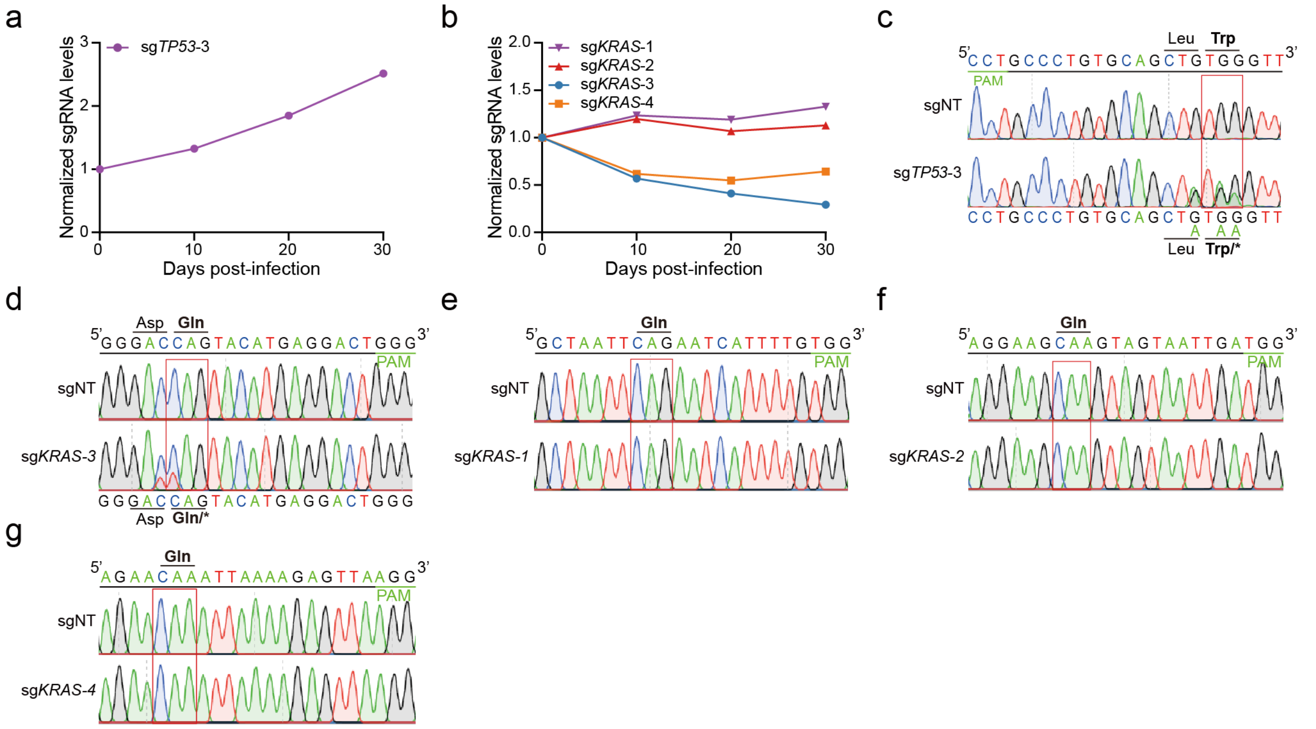

Supplementary Fig. 4 Validation of FNLS-BE3 base editor-based screening in A549 cells. a Relative abundance of sgRNA targeting *TP53*-3 locus for BE3 mediated CRISPR-STOP (n = 2 biologically independent samples). Data are presented as means. **b** Relative abundance of sgRNA targeting *KRAS* for BE3 mediated CRISPR-STOP (n = 2 biologically independent samples). Data are presented as means. **c** Representative sequence chromatogram of FNLS-BE3 treated A549 cells at *TP53-3* locus. **d-g** Representative sequence chromatogram of FNLS-BE3 treated A549 cells at *KRAS-3* (**d**)**,** *KRAS-1* (**e**), *KRAS-2* (**f**), and *KRAS-4* (**g**) loci.

Source data are provided as a Source Data file.

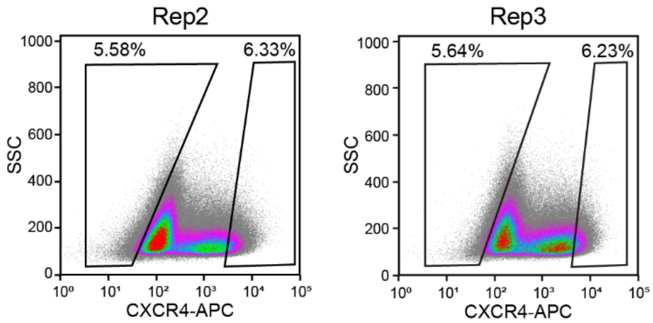

Supplementary Fig. 5 The other two replicates of FNLS-ABE7.10(AW) base editor-based screening in H1 hESCs. CXCR4 FACS gating strategy of sorting CXCR4^{+} and CXCR4^{-} populations for the other two replicates. Rep, replicate.

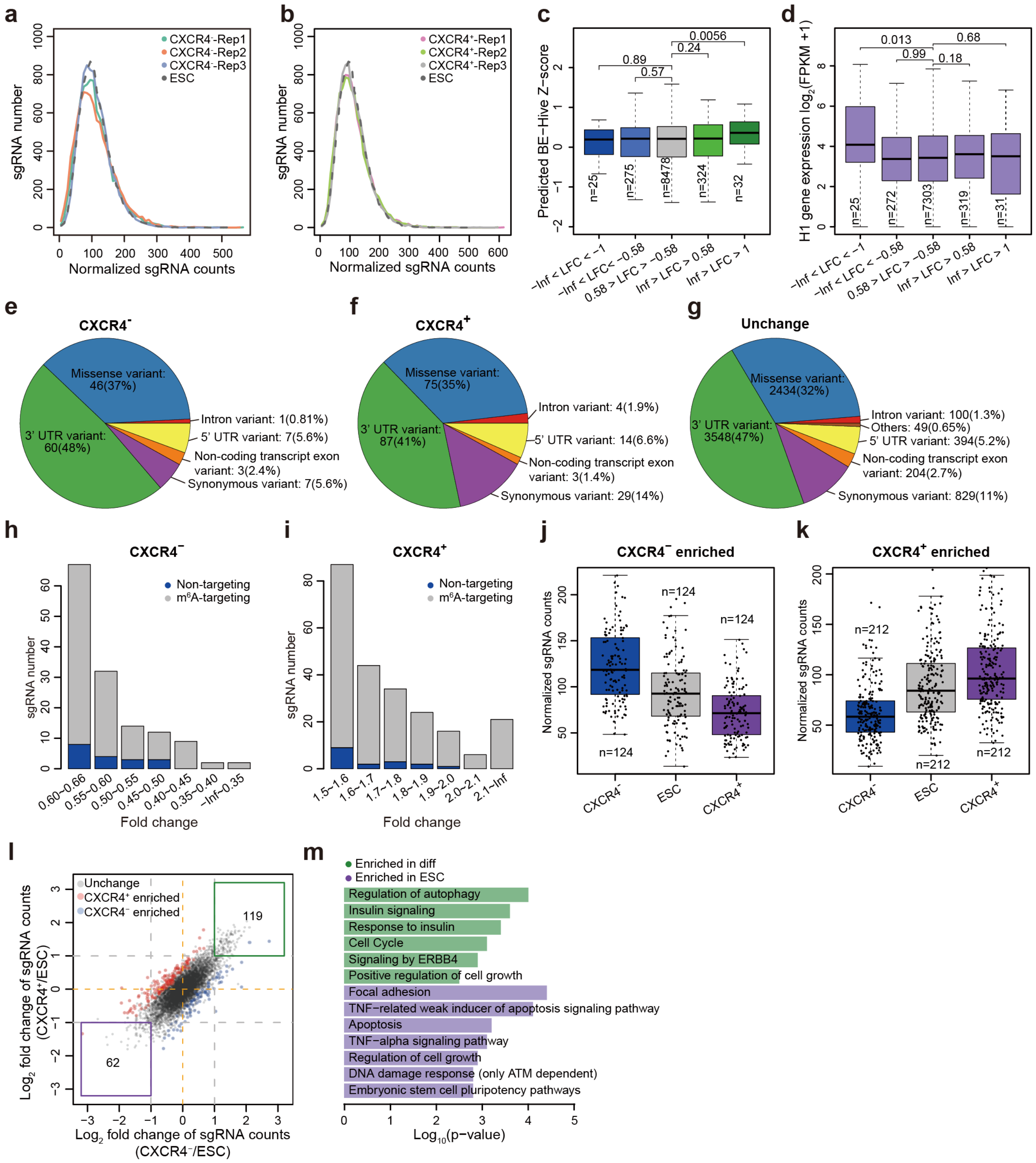

Supplementary Fig. 6 Analyses of FNLS-ABE7.10(AW) base editor-based functional screening of m^{6}A sites in H1 hESCs. a, b Distribution of normalized sgRNA counts in CXCR4$^-$ (**a**) and CXCR4$^+$ (**b**) respectively. **c** Box plot showing comparing the predicated BE-hive score of sgRNA stratified by log2 fold-changes of normalized sgRNA counts between CXCR4$^-$ and CXCR4$^+$. *P* values of pairwise comparisons are indicated (Two-tailed Wilcoxon test). The central horizontal line in the box indicates the median and the boundaries of the box indicate the first and third quartiles, and whiskers define the 1.5× interquartile range. **d** Box plot comparing the expression level of H1 cells for genes targeted by the

sgRNAs stratified by log2 fold-changes of normalized sgRNA counts between CXCR4$^-$ and CXCR4$^+$. *P* values of pairwise comparisons are indicated (Two-tailed Wilcoxon test). The central horizontal line in the box indicates the median and the boundaries of the box indicate the first and third quartiles, and whiskers define the 1.5× interquartile range. **e-g** Pie chart displaying the fractions of different annotations for the sgRNAs overrepresented in CXCR4$^-$ (**e**), CXCR4$^+$ (**f**) population, and unenriched sgRNAs (**g**) respectively. **h**, **i** Bar plots showing the frequencies of m^{6}A-targeting and non-targeting sgRNAs stratified by fold-change of normalized sgRNA counts (CXCR4$^+$/CXCR4$^-$) for sgRNAs overrepresented in CXCR4$^-$ (**h**) and CXCR4$^+$ (**i**) respectively. **j, k** Box plots comparing the normalized counts of CXCR4$^-$ (**j**) and CXCR4$^+$ (**k**) enriched sgRNAs in CXCR4$^-$, CXCR4$^+$ and ESC cells respectively. The central horizontal line in the box indicates the median and the boundaries of the box indicate the first and third quartiles, and whiskers define the 1.5× interquartile range. **l** Scatter plot comparing the log2 fold change of normalized counts for CXCR4$^-$ and CXCR4$^+$ relative to hESCs, the sgRNAs enriched or depleted in both of CXCR4$^-$ and CXCR4$^+$ are numbered and indicated by colored boxes. The significantly enriched sgRNAs in CXCR4$^-$ and CXCR4$^+$ are colored red and blue respectively. **m** GO enrichment of the genes targeted by the sgRNAs enriched or depleted in both CXCR4$^-$ and CXCR4$^+$ respectively.

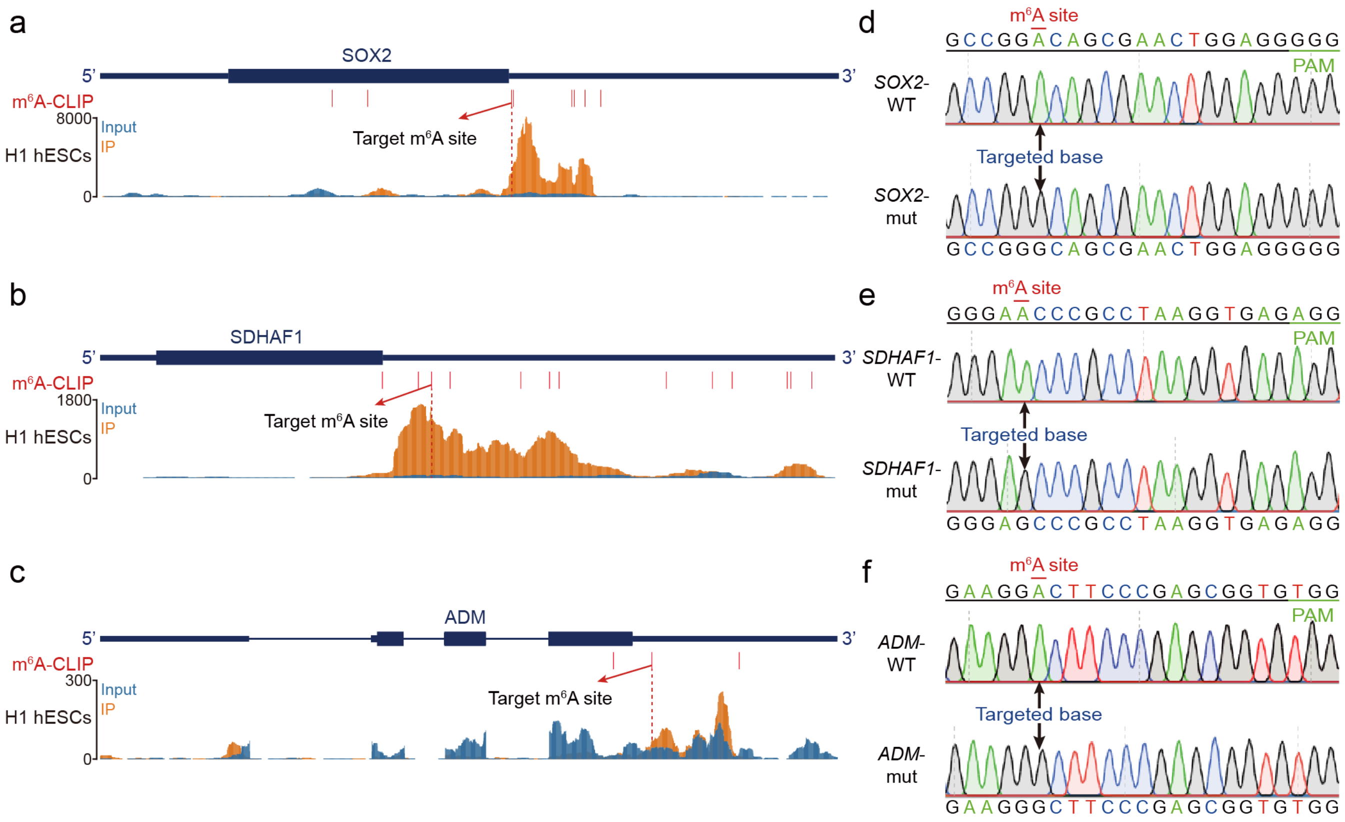

Supplementary Fig. 7 Track demonstration and Sanger sequencing validation of the ABE targetable m^{6}A sites on *SOX2, SDHAF2,* and *ADM* genes in hESC. a-c UCSC genome browser tracks of m^{6}A-seq signals in H1 hESCs and m^{6}A-CLIP-seq signals in A549 cells at the *SOX2* (**a**), *SDHAF1* (**b**), and *ADM* (**c**) loci. **d-f** Representative sequence chromatogram of *SOX2*-mut (**d**), *SDHAF1*-mut (**e**), and *ADM*-mut (**f**) homozygous mutant hESCs monoclonal.

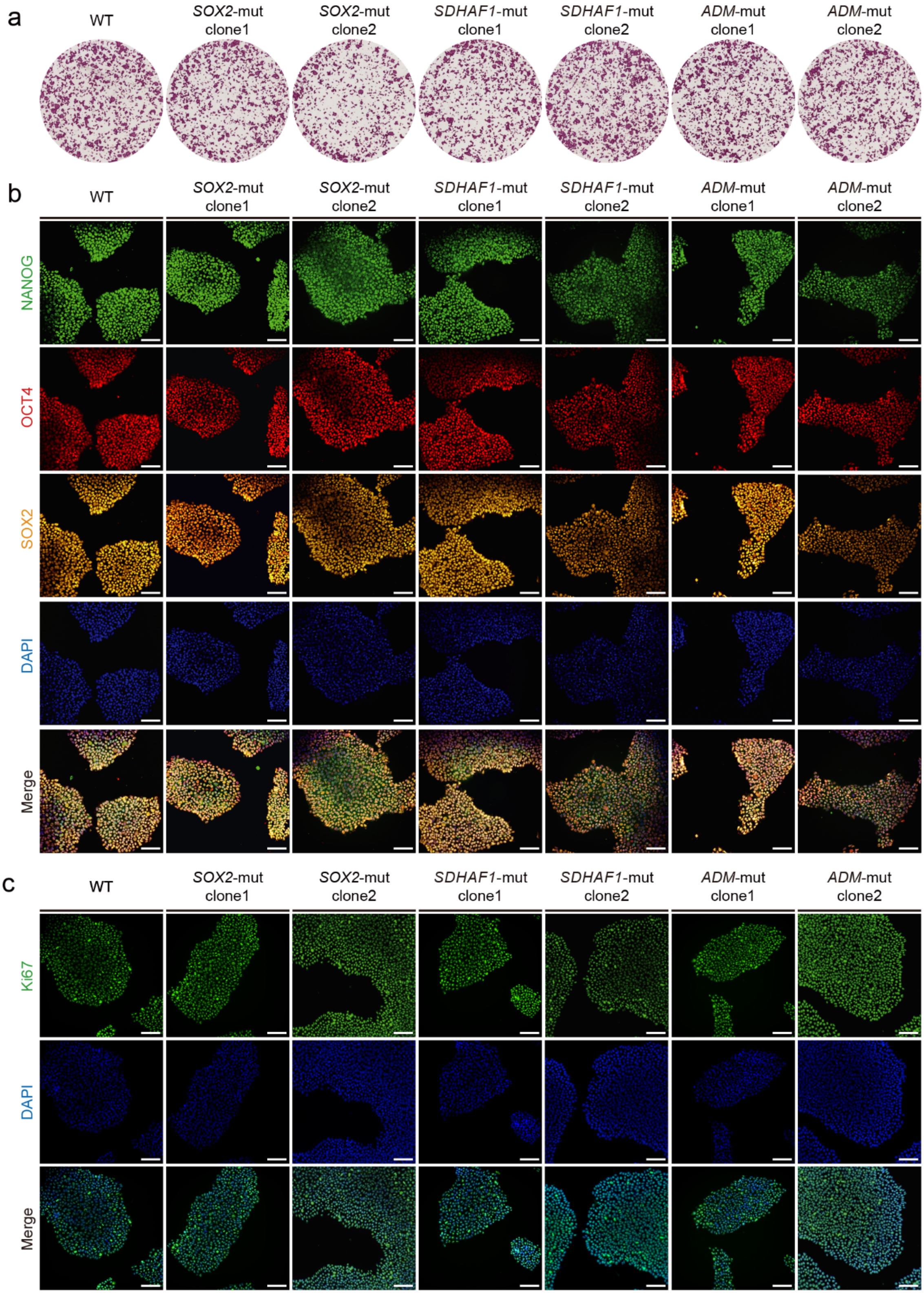

Supplementary Fig. 8 Characterization of mutant hESCs. a Alkaline phosphatase staining of each mutant hESCs. **b** Immunofluorescence analyses of the pluripotency markers NANOG, OCT4, and SOX2 in each mutant hESCs. **c** Immunofluorescence analyses of the proliferation marker Ki67 in each mutant hESCs. Scale bars, 100 µm.

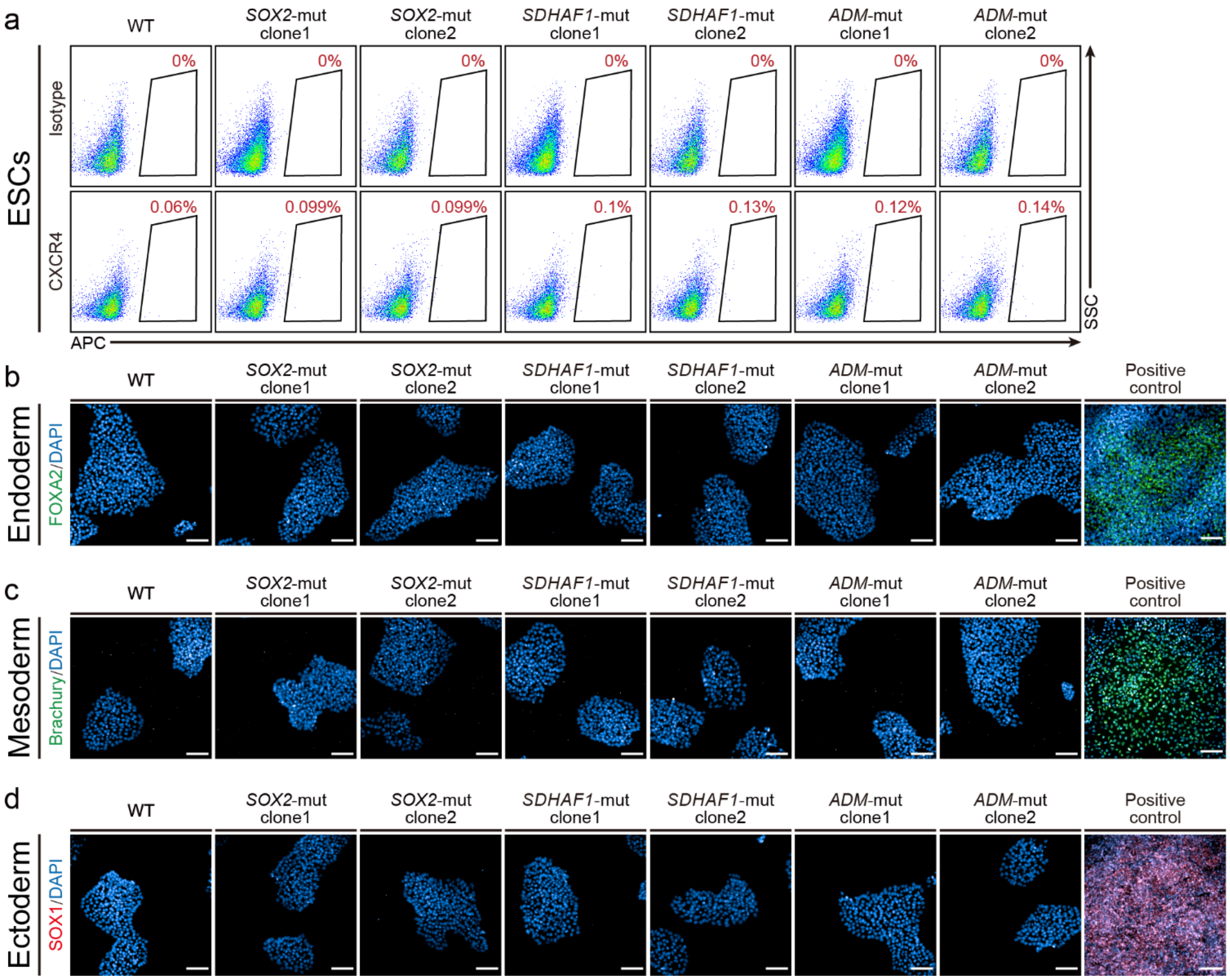

Supplementary Fig. 9 Multiple analyses of key germ-layer markers of each hESCs cell line. a Representative flow cytometry analysis of isotype and CXCR4 staining in WT, *SOX2*-mut, *SDHAF1*-mut, and *ADM*-mut hESCs. **b-d** Representative immunofluorescence analyses of endoderm marker FOXA2 (**b**), mesoderm marker Brachury (**c**), and ectoderm marker SOX1 (**d**), in WT, *SOX2*-mut, *SDHAF1*-mut, and *ADM*-mut hESCs. Scale bars, 100μm.

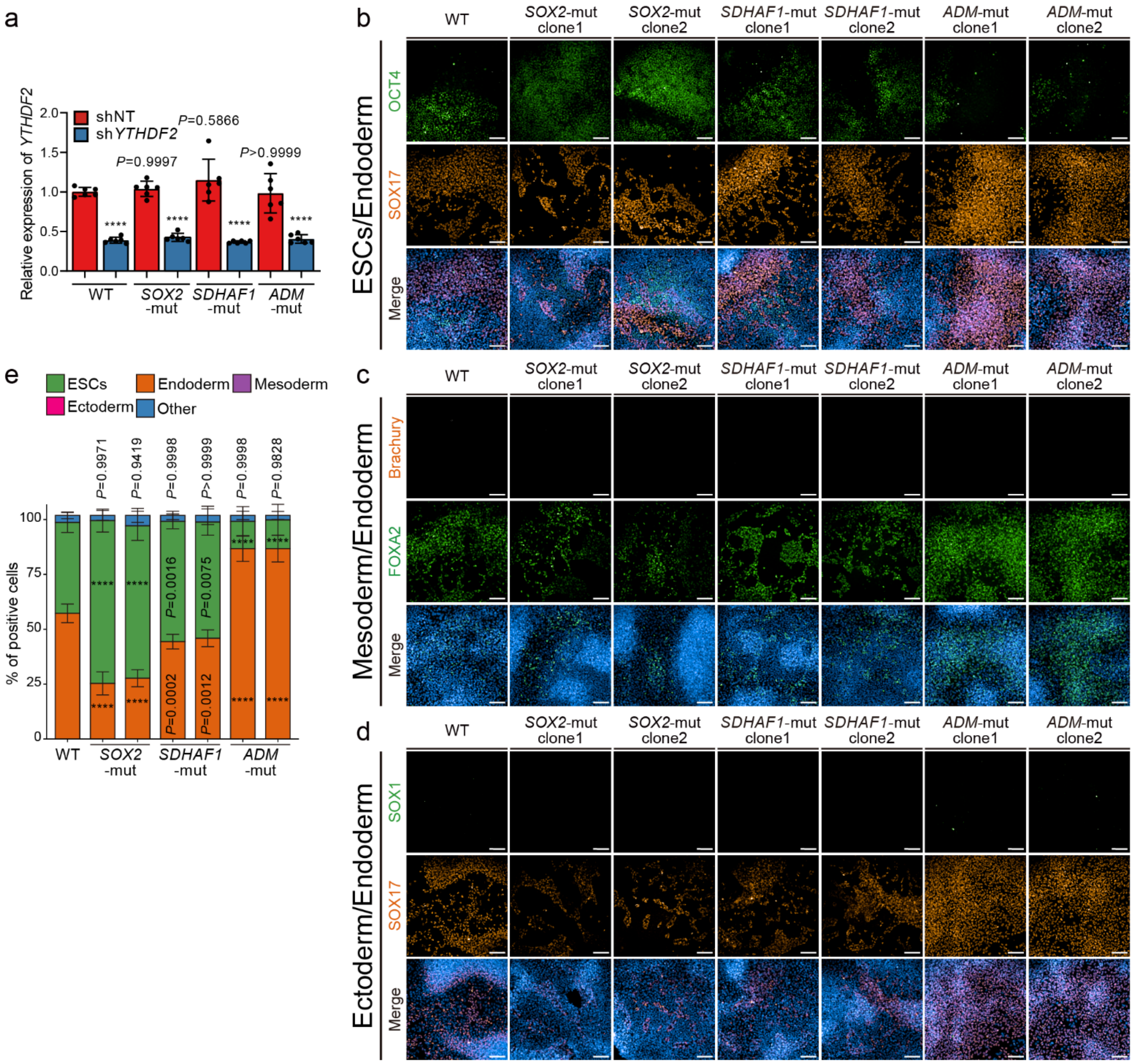

Supplementary Fig. 10 Immunofluorescence analyses of key germ-layer markers in cultures at day 3 of DE differentiation. a shRNA knockdown efficiency of *YTHDF2* in WT, *SOX2*-mut, *SDHAF1*-mut, and *ADM*-mut hESCs (n = 6 biologically independent samples), measured by quantitative RT-PCR and relative expression level was calculated vs WT with shNT. NT, non-targeting. *P*-values were calculated vs WT with shNT and **** indicate *P*<0.0001 (One-way ANOVA with Tukey's post hoc test). **b-e** Representative (**b-d**) and quantitative (**e**) immunofluorescence analyses the co-expression of endoderm marker FOXA2/SOX17 with ESCs marker OCT4 (**b**), mesoderm marker Brachury (**c**), and ectoderm marker SOX1 (**d**) in cultures derived from WT, *SOX2*-mut, *SDHAF1*-mut, and *ADM*-mut hESCs at day 3 of DE differentiation (n = 6 biologically independent samples). *P*-values were calculated vs each portion in WT and **** indicate *P*<0.0001 (One-way ANOVA with Tukey's post hoc test). Scale bars, 100 µm. Data are presented as means ± SD.

Source data are provided as a Source Data file.

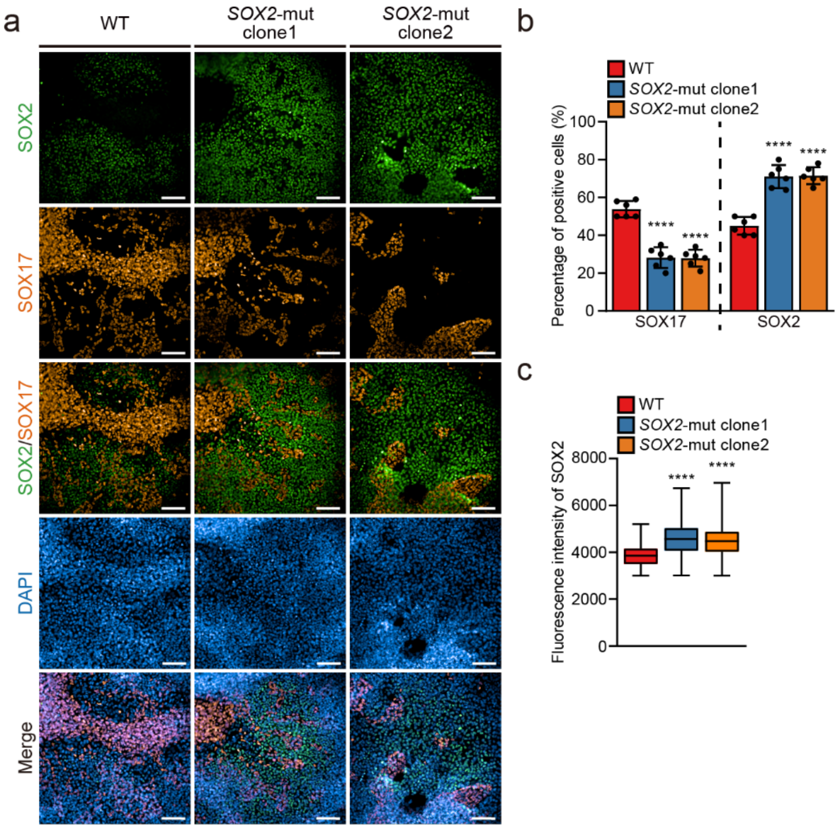

Supplementary Fig. 11 Site-specific m^{6}A elimination resulted in the continuous increase of SOX2 protein expression. a, b Representative (**a**) and quantitative (**b**, n = 6 biologically independent samples) immunofluorescence analyses the co-expression of endoderm marker SOX17 and SOX2 in cultures derived from WT and *SOX2*-mut hESCs at day 3 of DE differentiation. *P*-values were calculated vs WT and **** indicate P<0.0001 (One-way ANOVA with Tukey's post hoc test). Scale bars, 100 μm. Data are presented as means ± SD. **c** Mean fluorescence intensity of SOX2 in cultures derived from WT and *SOX2*-mut hESCs at day 3 of DE differentiation (n = 1016, 1602, and 1362 cells for WT, *SOX2*-mut clone1, and *SOX2*-mut clone2 respectively). *P*-values were calculated vs WT and **** indicate P<0.0001 (One-way ANOVA with Tukey's post hoc test). The central horizontal line in the box indicates the median and the boundaries of the box indicate the first and third quartiles, and whiskers define the 1.5× interquartile range. Source data are provided as a Source Data file.

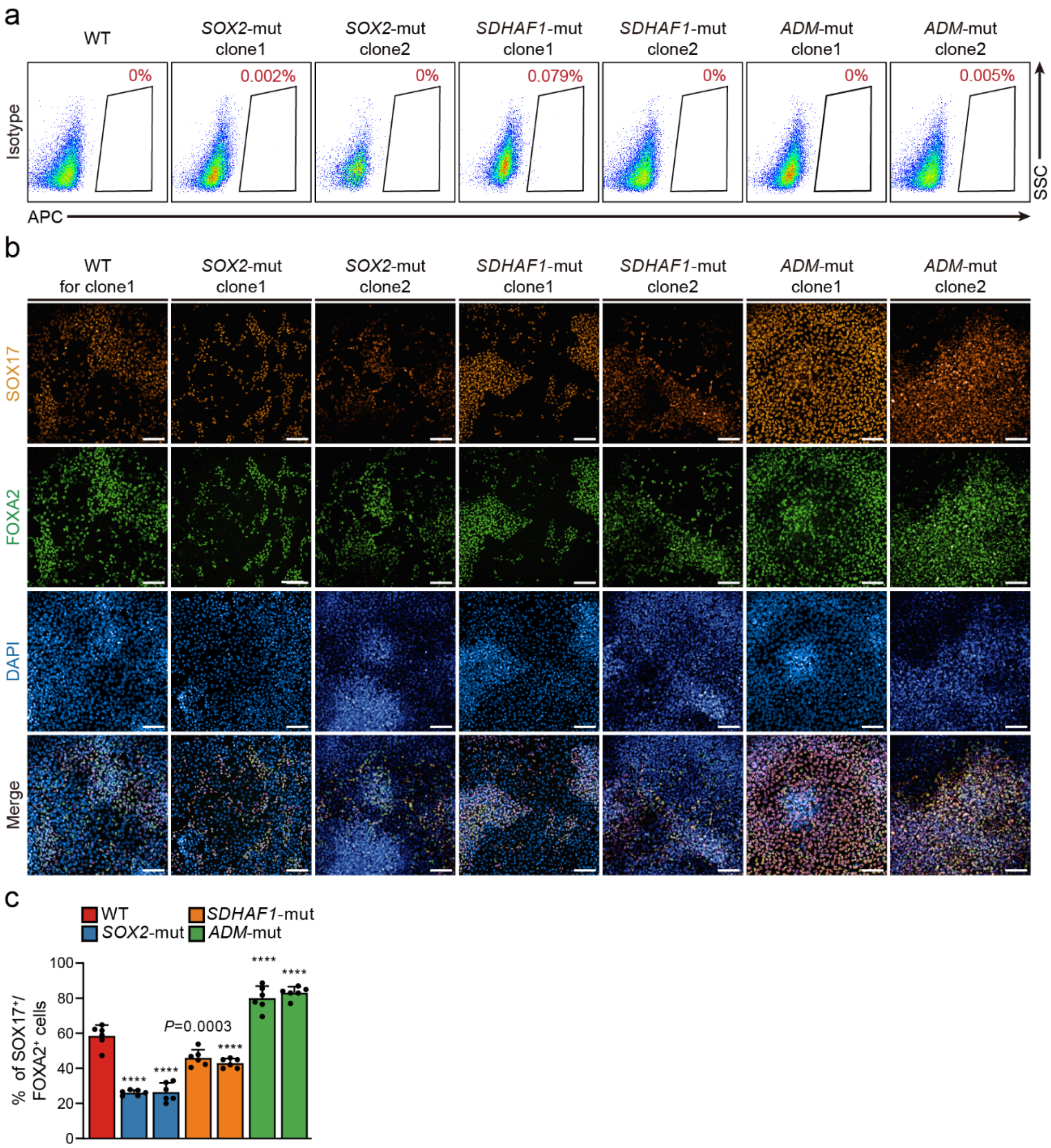

Supplementary Fig. 12 Immunofluorescence analyses of DE gene expressions during differentiation. **a** Representative flow cytometry analysis of isotype staining in cultures derived from WT, *SOX2*-mut, *SDHAF1*-mut, and *ADM*-mut hESCs at day 3 of DE differentiation. **b, c** Representative (**b**) and quantitative (**c**) immunofluorescence analyses of DE markers SOX17 and FOXA2 in cultures derived from WT, *SOX2*-mut, *SDHAF1*-mut, and *ADM*-mut hESCs at day 3 of DE differentiation (n = 6 biologically independent samples). *P*-values were calculated vs WT and **** indicate *P*<0.0001 (One-way ANOVA with Tukey's post hoc test). Scale bars, 100 μm. Data are presented as means ± SD.

Source data are provided as a Source Data file.

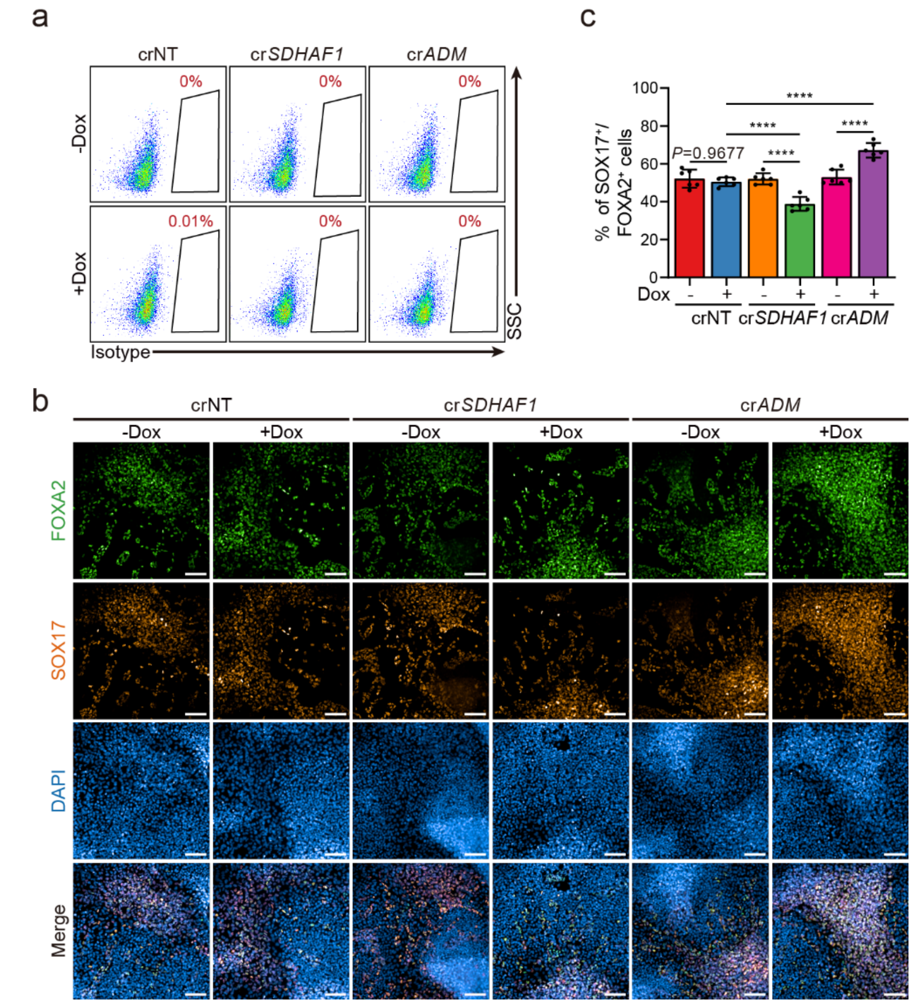

Supplementary Fig. 13 TRME editor induces site-specific m^{6}A demethylation and affects DE differentiation efficiency in hESCs. a Representative flow cytometry analysis of isotype staining in cultures derived from hESCs with or without induced site-specific m^{6}A demethylation by TRME editor at day 3 of DE differentiation. Dox, doxycycline. **b, c** Representative (**b**) and quantitative (**c**, n = 6 biologically independent samples) immunofluorescence analyses of DE markers SOX17 and FOXA2 protein expression in cultures derived from hESCs with or without induced site-specific m^{6}A demethylation by TRME editor at day 3 of DE differentiation. NT, non-targeting; Dox, doxycycline. **** indicate P<0.0001 (One-way ANOVA with Tukey's post hoc test). Scale bars, 100 μm. Data are presented as means ± SD.

Source data are provided as a Source Data file.

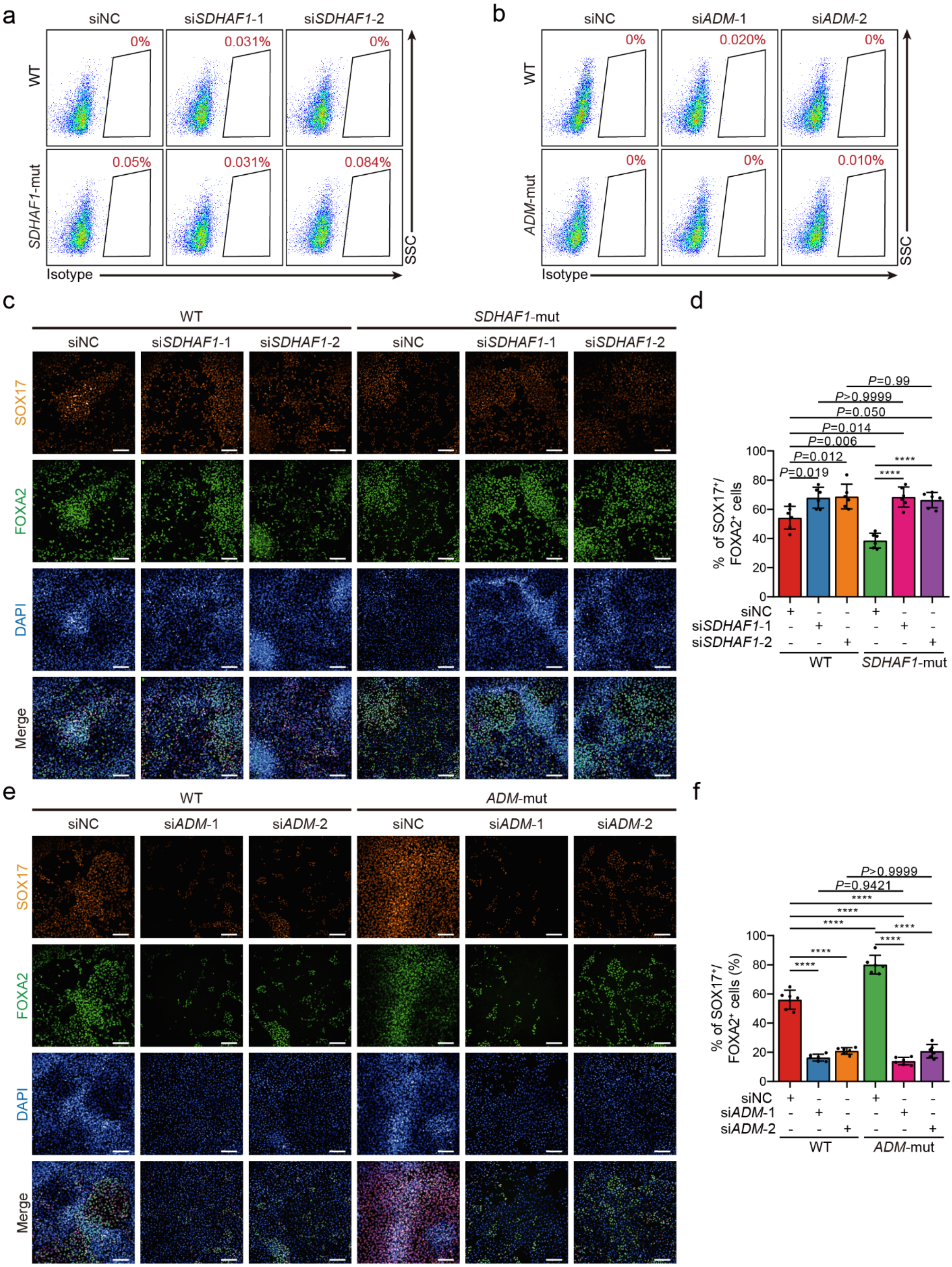

Supplementary Fig. 14 Immunofluorescence analyses of DE markers after *SDHAF1* and *ADM* knockdown. a, b Representative flow cytometry analyses of isotype staining in cultures derived from WT or each mutant hESCs that received either NC or *SDHAF1*-targeting siRNAs (**a**)/*ADM*-targeting siRNAs (**b**) at day 3 of DE differentiation. NT, non-targeting; Dox, doxycycline. **c, d, e, f** Representative (**c, e**) and

quantitative (**d, f**) immunofluorescence analyses of DE marker SOX17 and FOXA2 expression in cultures derived from WT or each mutant hESCs that received either NC or *SDHAF1*-targeting siRNAs (**c, d**)/*ADM*-targeting siRNAs (**e, f**) at day 3 of DE differentiation (n = 6 biologically independent samples). **** indicate P<0.0001 (One-way ANOVA with Tukey's post hoc test). Scale bars, 100μm. Data are presented as means ± SD.

Source data are provided as a Source Data file.

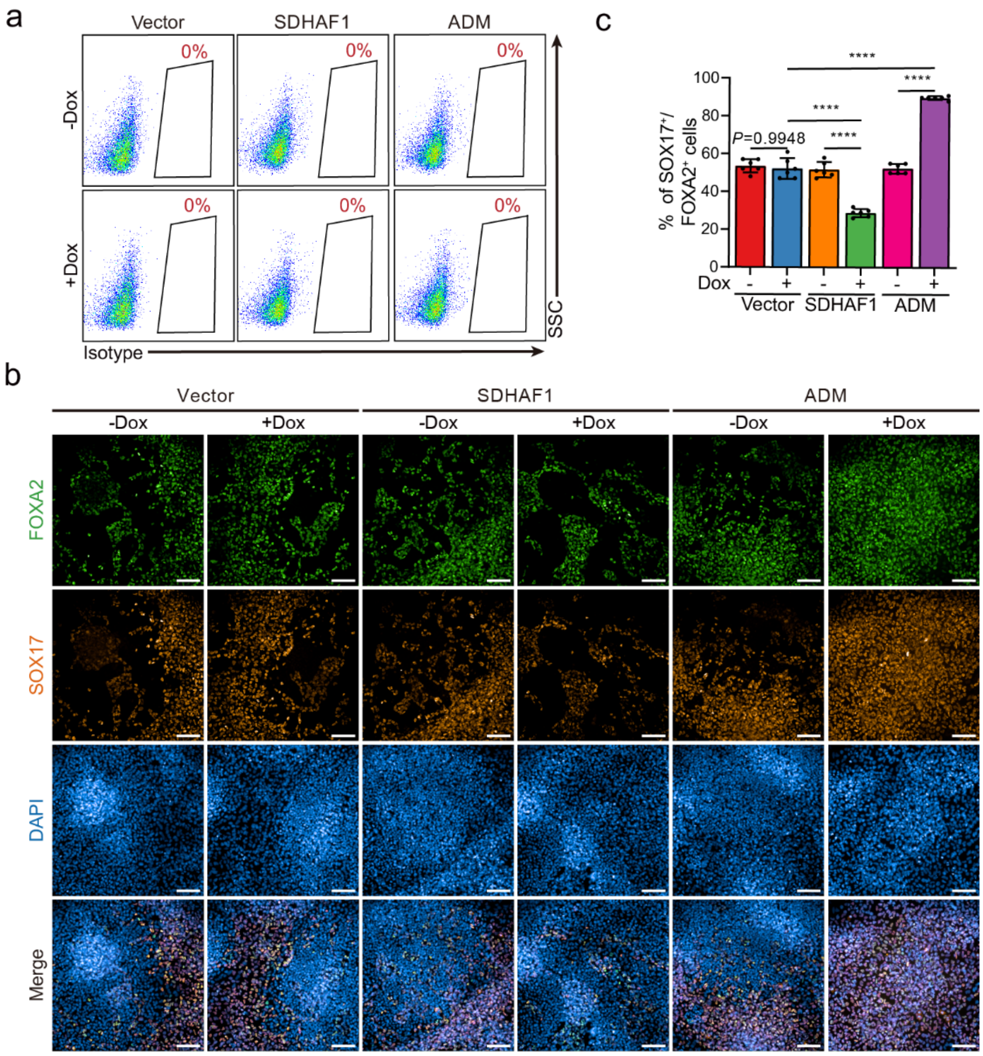

Supplementary Fig. 15 Immunofluorescence analyses of DE markers after *SDHAF1* and *ADM* overexpression. a Representative flow cytometry analysis of isotype staining in cultures derived from WT hESCs or hESCs that overexpressed *SDHAF1*/*ADM* at day 3 of DE differentiation. OE, Overexpression; Dox, doxycycline. **b, c** Representative (**b**) and quantitative (**c**) immunofluorescence analyses of DE marker SOX17 and FOXA2 expression in cultures derived from WT hESCs or hESCs that overexpressed either *SDHAF1* or *ADM* at day 3 of DE differentiation (n = 6 biologically independent samples). **** indicate $P<0.0001$ (One-way ANOVA with Tukey's post hoc test). Scale bars, 100μm. Data are presented as means ± SD.

Source data are provided as a Source Data file.

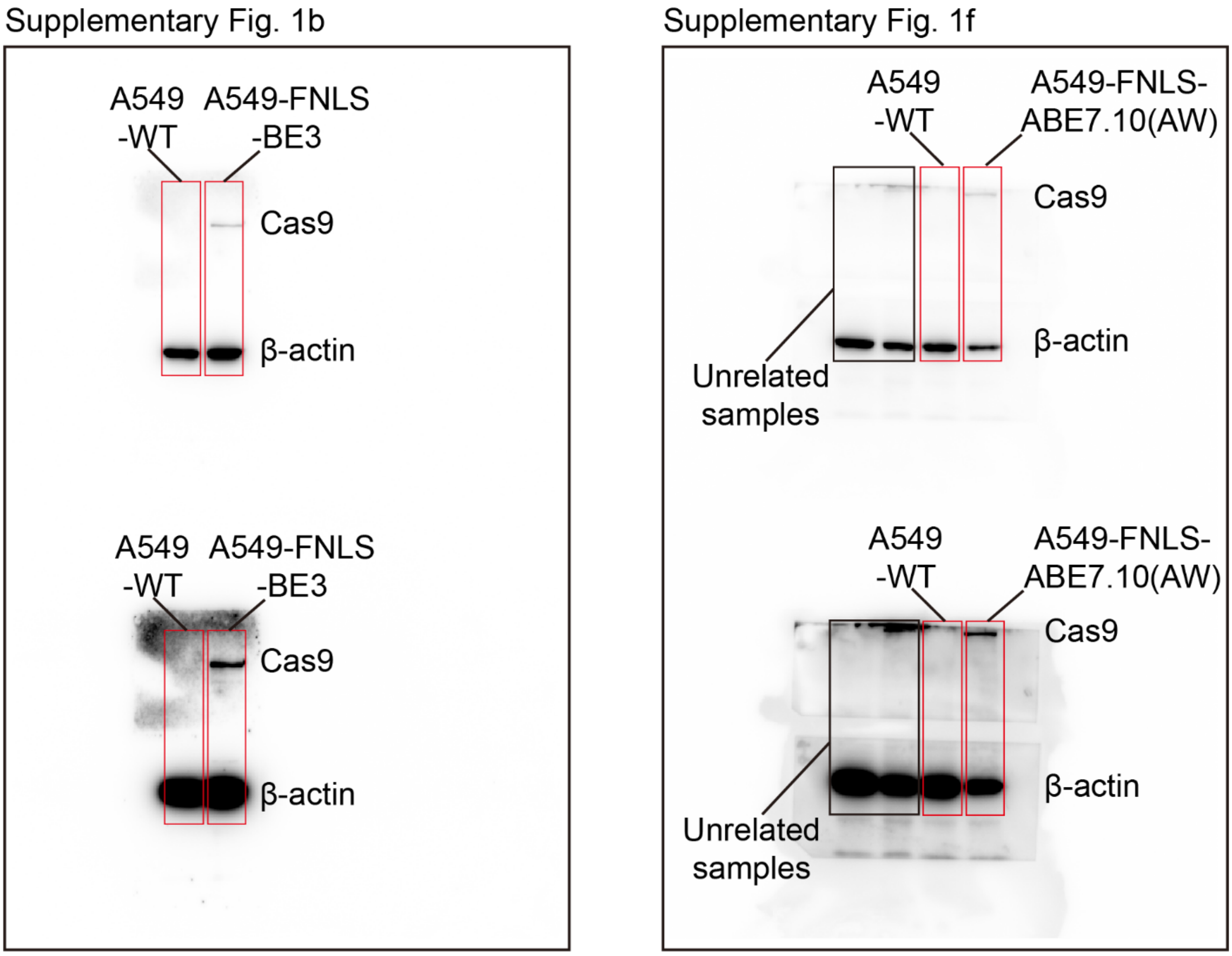

Fig. 16 Original un-cropped images of western blots.

Supplementary Table 1 sgRNAs, shRNAs, or crRNAs sequences used in this study(sequences are from 5' to 3')

Locus	**Sequence**
sg*HEK4*	GCACTGCGGCTGGAGGTGG
sg*METTL3*-1	AAGCAGCTGGACTCTCTGC
sg*EEF2*-2	TAGGGACTTAATGGGCCGGT
sg*NEAT1*	TTAGACTGGGGATATATTAG
sg*MYC*-1	TTCTTcCAGATATCCTCGCT
sg*MYC*-2	ATCcAGGACTGTATGTGGAG
sg*MYC*-3	AGCCGCTcCACATACAGTCC
sg*MYC*-4	AGAGGcAGGCTCCTGGCAAA
sg*KRAS*-1	GCTAATTcAGAATCATTTTG
sg*KRAS*-2	AGGAAGcAAGTAGTAATTGA
sg*KRAS*-3	GGGACcAGTACATGAGGACT
sg*KRAS*-4	AGAAcAAATTAAAAGAGTTA
sg*TP53*-3	AACcCACAGCTGCACAGGGC
sg*SOX2*	GGCCGGaCAGCGAACTGGAG
sg*SDHAF1*	GGGAaCCCGCCTAAGGTGAG
sg*ADM*	GAAGGaCTTCCCGAGCGGTG
sh*YTHDF2*	GATGGATTAAACGATGATGAT
cr*SDHAF1*	GGGTTCCCCAGTCCCCCCATGCAGTCAG
cr*ADM*	AAGTCCTTCGTCCCGGGAGGCACCAGCG
crNT	TAGATTGCTGTTCTACCAAGTAATCCAT

Supplementary Table 2 Primer sequences used for target amplification(sequences are from 5' to 3')

Locus	***Forward***	***Reverse***
sg*HEK4*	TACTGCGTGGAGACAGACCA	TTTCAACCCGAACGGAGACA
sg*METTL3*-1	CTTCCAAGAAAGCGCGACAC	GAGGGAACAAAGCGCCAAAG
sg*EEF2*-2	TGACAGGACTTTCCTTCTGCC	CATCTACCCGCGTGTTCCTT
sg*NEAT1*	TCTAAATTGAGCCTCCGGTCA	GGTGTACCCACCATTCCCTT
sg*MYC*-1	CTCCGAGATAGCAGGGGACT	CGGGTCGCAGATGAAACTCT
sg*MYC*-2/3	CATCAGCACAACTACGCAGC	GTCGTTTCCGCAACAAGTCC
sg*MYC*-4	AGTAAGGGTGGCTGGCTAGA	GCTGGTGCATTTTCGGTTGT
sg*KRAS*-1	TGGACCCTGACATACTCCCA	AGGGTGTGCTACAGGGTGTA
sg*KRAS*-2/3	GTGTCTGTATCCTTGCTAACAC	TCCACTGCTCTAATCCCCCA
sg*KRAS*-4	AGCAATGCCCTCTCAAGAGAC	TGTAGTAACTTCAGTGTTGCC
sg*TP53*-3	AGCTGCTCACCATCGCTATC	GCCAACTCTCTCTAGCTCGC
sg*SOX2*	ATGGGTTCGGTGGTCAAGTC	TTCCTGCAAAGCTCCTACCG
sg*ADM*	CTACCGCCAGAGCATGAACA	TCCTCTCCTTAAGCCTGGCA
sg*SDHAF1*	CATCGAGTACCTGTACCGCC	CTTGAGGAGCCAGAAGTGGG

Supplementary Table 3 siRNA sequences used in this study (sequences are from 5' to 3')

Name	***Sequences***
si*ADM*-1	GCGUCGGAGUUUCGAAAGA
si*ADM*-2	GGUGUCUUCUAAGCCACAA
si*SDHAF1*-1	UGCGCAUCGAGUACCUGUA
si*SDHAF1*-2	GACGGCGACAGUCCAAGGA

Supplementary Table 4 Primers used in the SELECT assays (sequences are from 5’ to 3’)

Locus	**Up**	**Down**
NEAT1-m^{6}A-c.1793	TAGCCAGTACCGTAGTGCGTGAATCTGCTTCCTCTAATATATCCCCAG	CTAAGGCATTTAAAATTAAACAGCTCTTCCAGAGGCTGAGTCGCTGCAT
NEAT1-A-c.1800	TAGCCAGTACCGTAGTGCGTGTTGACAATCTGCTTCCTCTAATATA	CCCCAGTCTAAGGCATTTAAAATTACAGAGGCTGAGTCGCTGCAT
EEF2-m^{6}A-c.*226	TAGCCAGTACCGTAGTGCGTGCTCCCCGACCGGCCCATTAAG	CCCTACTAAGAGGGCGTGTCTGCAGAGGCTGAGTCGCTGCAT
EEF2-A	TAGCCAGTACCGTAGTGCGTGCAGGAAGGGCCGCCTACAAT	TGTCCAGGAAGTTGTCCAGGGCCAGAGGCTGAGTCGCTGCAT
SOX2-m^{6}A-c.*8	TAGCCAGTACCGTAGTGCGTGTCTCCCCCCTCCAGTTCGCTG	CCGGCCCTCACATGTGTGAGACAGAGGCTGAGTCGCTGCAT
SOX2-A	TAGCCAGTACCGTAGTGCGTGTCACATGTGTGAGAGGGGCAG	GTGCCGTTAATGGCCGTGCCCAGAGGCTGAGTCGCTGCAT
SDHAF1-m^{6}A-c.*76	TAGCCAGTACCGTAGTGCGTGAGACCTCTCACCTTAGGCGGG	TCCCCAGTCCCCCCATGCAGCAGAGGCTGAGTCGCTGCAT
SDHAF1-A	TAGCCAGTACCGTAGTGCGTGTCGCCAAGGAGGAGTCTCTGACA	CCCCAATTCGTCAAGCTAGTCTCCAGAGGCTGAGTCGCTGCAT
ADM-m^{6}A-c.*68	TAGCCAGTACCGTAGTGCGTGGTCCCCACACCGCTCGGGAAG	CCTTCGTCCCGGGAGGCACCCAGAGGCTGAGTCGCTGCAT
ADM-A	TAGCCAGTACCGTAGTGCGTGCGGGATGCTTGCGCGACTAT	CCTTGTACCATGGGCGCCTAAACAGAGGCTGAGTCGCTGCAT
qPCR for SELECT	ATGCAGCGACTCAGCCTCTG	TAGCCAGTACCGTAGTGCGTG

Supplementary Table 5 RT-qPCR primer sequences used in this study (sequences are from 5' to 3')

Gene	Forward	Reverse
GAPDH	GTCTCCTCTGACTTCAACAGCG	ACCACCCTGTTGCTGTAGCCAA
18S	ACCCGTTGAACCCCATTCGTGA	GCCTCACTAAACCATCCAATCGG
SOX2	GCTACAGCATGATGCAGGACCA	TCTGCGAGCTGGTCATGGAGTT
SDHAF1	GCGCATCGAGTACCTGTAC	TGGGGTTCCTTGGACTGT
ADM	GACATGAAGGGTGCCTCTCGAA	CCTGGAAGTTGTTCATGCTCTGG
SOX17	GTGGACCGCACGGAATTTG	GGAGATTCACACCGGAGTCA
FOXA2	ATTGCTGGTCGTTTGTTGTG	CCTCGGGCTCTGCATAGTAG
CXCR4	ACTACACCGAGGAAATGGGCT	CCCACAATGCCAGTTAAGAAGA
GATA4	CGACACCCCAATCTCGATATG	GTTGCACAGATAGTGACCCGT
GATA6	CTCAGTTCCTACGCTTCGCAT	GTCGAGGTCAGTGAACAGCA
HNF1B	ACCAAGCCGGTCTTCCATACT	GGTGTGTCATAGTCGTCGCC
YTHDF2	GCGCATCGAGTACCTGTAC	TGGGGTTCCTTGGACTGT
SOX2-RIP	GCCATTAACGGCACACTGC	CCCTCCCATTTCCCTCGTTT
SDHAF1-RIP	CGAACTCGCTCGATGGCG	ACATCCCCAATTCGTCAAGCTA
ADM-RIP	AGGATTTAGGCGCCCATGGTA	CACACCGCTCGGGAAGT
