# Supplementary material for: Parallel functional assessment of m6A sites in human endodermal differentiation with base editor screens
Source: Nat Commun. 2022 Jan 25;13:478. doi: 10.1038/s41467-022-28106-0 (PMC8789821; doi:10.1038/s41467-022-28106-0)
Supplement: Supplementary file 2 — Reporting Summary [file 41467_2022_28106_MOESM2_ESM.pdf]

nature portfolio

Corresponding author(s): Jinkai Wang & Nan Cao

Last updated by author(s): Nov 2, 2021

Reporting Summary

Nature Portfolio wishes to improve the reproducibility of the work that we publish. This form provides structure for consistency and transparency in reporting. For further information on Nature Portfolio policies, see our Editorial Policies and the Editorial Policy Checklist.

Statistics

For all statistical analyses, confirm that the following items are present in the figure legend, table legend, main text, or Methods section.

n/a	Confirmed	
☐	☒	The exact sample size (n) for each experimental group/condition, given as a discrete number and unit of measurement
☐	☒	A statement on whether measurements were taken from distinct samples or whether the same sample was measured repeatedly
☐	☒	The statistical test(s) used AND whether they are one- or two-sided<br>*Only common tests should be described solely by name; describe more complex techniques in the Methods section.*
☒	☐	A description of all covariates tested
☐	☒	A description of any assumptions or corrections, such as tests of normality and adjustment for multiple comparisons
☐	☒	A full description of the statistical parameters including central tendency (e.g. means) or other basic estimates (e.g. regression coefficient) AND variation (e.g. standard deviation) or associated estimates of uncertainty (e.g. confidence intervals)
☒	☐	For null hypothesis testing, the test statistic (e.g. F, t, r) with confidence intervals, effect sizes, degrees of freedom and P value noted<br>*Give P values as exact values whenever suitable.*
☒	☐	For Bayesian analysis, information on the choice of priors and Markov chain Monte Carlo settings
☒	☐	For hierarchical and complex designs, identification of the appropriate level for tests and full reporting of outcomes
☒	☐	Estimates of effect sizes (e.g. Cohen's d, Pearson's r), indicating how they were calculated

Our web collection on statistics for biologists contains articles on many of the points above.

Software and code

Policy information about availability of computer code

Data collection	FlowJo Software (FlowJo LCC), BE-Hive algorithm, Operetta CLS system (Perkin Elmer).
Data analysis	GraphPad Prism (v8.0), R(4.0.0), MAGeCK (0.5), Cutadapt (3.4), Operetta CLS system (Perkin Elmer), SnapGene (GSL Biotech LLC), Metascape (3.5) and VEP (release 104). In house codes have been deposited to Github (https://github.com/ZJRen9/CRISPR_screen_effective_m6ASite).

For manuscripts utilizing custom algorithms or software that are central to the research but not yet described in published literature, software must be made available to editors and reviewers. We strongly encourage code deposition in a community repository (e.g. GitHub). See the Nature Portfolio guidelines for submitting code & software for further information.

Data

Policy information about availability of data

All manuscripts must include a data availability statement. This statement should provide the following information, where applicable:

- Accession codes, unique identifiers, or web links for publicly available datasets
- A description of any restrictions on data availability
- For clinical datasets or third party data, please ensure that the statement adheres to our policy

Source data are provided with this paper. Inforamtions of the designed sgRNA library are provided in the Supplementary Data 1. The sgRNA counts data generated in this study are provided in the Supplementary Data 2 and 3. The raw data generated in this study have been deposited in the GEO database under accession number GSE179980 (https://www.ncbi.nlm.nih.gov/geo/query/acc.cgi?acc=GSE179980). The original un-cropped images of western blots in this study are provided in the Supplementary Fig. 16.

Field-specific reporting

Please select the one below that is the best fit for your research. If you are not sure, read the appropriate sections before making your selection.

☒ Life sciences ☐ Behavioural & social sciences ☐ Ecological, evolutionary & environmental sciences

For a reference copy of the document with all sections, see nature.com/documents/nr-reporting-summary-flat.pdf

Life sciences study design

All studies must disclose on these points even when the disclosure is negative.

Sample size	In vitro studies were repeated at least three times independently to enable statistical analyses.
Data exclusions	In qPCR experiments, samples that had abnormal dissociation curves or non-detected CT values, which means the values were invalid according to a standard qPCR procedure, were excluded.
Replication	In all experiments at least 3 biological replications were performed unless otherwise indicated in the figure legends for technical or economic reason.
Randomization	For in vitro experiments, samples were allocated into experimental groups randomly.
Blinding	Although the investigators responsible for group allocation were not blinded to the allocation scheme, they were blinded to group allocation during data collection, and the investigators responsible for analyses were blinded to the allocation scheme (i.e., non-identifying codes were used as sample designations).

Reporting for specific materials, systems and methods

We require information from authors about some types of materials, experimental systems and methods used in many studies. Here, indicate whether each material, system or method listed is relevant to your study. If you are not sure if a list item applies to your research, read the appropriate section before selecting a response.

Materials & experimental systems

n/a	Involved in the study	
☐	☒	Antibodies
☐	☒	Eukaryotic cell lines
☒	☐	Palaeontology and archaeology
☒	☐	Animals and other organisms
☒	☐	Human research participants
☒	☐	Clinical data
☒	☐	Dual use research of concern

Methods

n/a	Involved in the study	
☒	☐	ChIP-seq
☐	☒	Flow cytometry
☒	☐	MRI-based neuroimaging

Antibodies

Antibodies used

APC-conjugated isotype (Clone: eBM2a, Invitrogen, Cat# 17-4724-81, 1 μg per 10^6 cells)
APC-conjugated anti-CXCR4 (Clone: 12G5, Invitrogen, Cat#17-9999-42, 1 μg per 10^6 cells)
Cas9 (Clone: polyclonal, Diagenode, Cat# C15310258, 1:3000)
β-actin (Clone: unknown, 4A Biotech, Cat# 4ab080291, 1:1000)
SOX17 (Clone: polyclonal, R&D, Cat# AF1924, 1:200)
FOXA2 (Clone: D56D6, CST, Cat# 8186S, 1:200)
SOX2 (Clone: 9-9-3, Abcam, Cat# ab79351, 1:200)
NANOG (Clone: polyclonal, CST, Cat# 3580S, 1:200)
OCT4 (Clone: C-10, Santa Cruz, Cat# sc-5279, 1:200)
SOX1 (Clone: polyclonal, CST, Cat# 4194S, 1:200)
Brachury (Clone: polyclonal, R&D, Cat# AF2085, 1:100)
Ki67 (Clone:B56 , BD Biosciences, Cat#550609, 1:100)
YTHDF2 (Proteintech, 24744-1-AP, 5μg per RIP sample)
Secondary antibodies conjugated with Alexa-488, Alexa-555, Alexa-594or Alexa-647 (Cat# A21206, A21121, A21242, A21127, A11058; Invitrogen; 1:800).

Validation

Validation details of the antibodies are available on the manufacturers' websites:
APC-conjugated isotype (https://www.thermofisher.cn/cn/zh/antibody/product/Mouse-IgG2a-kappa-clone-eBM2a-Isotype-Control/17-4724-81)
APC-conjugated anti-CXCR4 (https://www.thermofisher.cn/cn/zh/antibody/product/CD184-CXCR4-Antibody-clone-12G5-

Monoclonal/17-9999-42)
Cas9 (https://www.diagenode.com/cn/documents/c15310258-datasheet-crispr-cas9-polyclonal-antibody)
β-actin (http://4abio.com/goods-1321486.html)
SOX17 (https://www.rndsystems.com/cn/products/human-sox17-antibody_af1924)
FOXA2 (https://www.cellsignal.cn/products/primary-antibodies/nanog-antibody/8186)
SOX2 (https://www.abcam.cn/sox2-antibody-9-9-3-ab79351.html)
NANOG (https://www.cellsignal.cn/products/primary-antibodies/nanog-antibody/3580)
OCT4 (https://www.scbt.com/p/oct-3-4-antibody-c-10)
SOX1 (https://www.cellsignal.cn/products/primary-antibodies/nanog-antibody/4194)
Brachury (https://www.rndsystems.com/cn/products/human-mouse-brachyury-antibody_af2085)
Ki67 (https://www.bdbiosciences.com/zh-cn/products/reagents/flow-cytometry-reagents/research-reagents/single-color-antibodies-ruo/purified-mouse-anti-ki-67.550609)
YTHDF2 (http://www.ptgcn.com/products/YTHDF2-Antibody-24744-1-AP.htm)
Secondary antibodies conjugated with Alexa-488 (https://www.thermofisher.cn/cn/zh/antibody/product/Donkey-anti-Rabbit-IgG-H-L-Highly-Cross-Adsorbed-Secondary-Antibody-Polyclonal/A-21206)
Secondary antibodies conjugated with Alexa-488 (https://www.thermofisher.cn/cn/zh/antibody/product/Goat-anti-Mouse-IgG1-Cross-Adsorbed-Secondary-Antibody-Polyclonal/A-21121)
Secondary antibodies conjugated with Alexa-647 (https://www.thermofisher.cn/cn/zh/antibody/product/Goat-anti-Mouse-IgG2b-Cross-Adsorbed-Secondary-Antibody-Polyclonal/A-21242)
Secondary antibodies conjugated with Alexa-555 (https://www.thermofisher.cn/cn/zh/antibody/product/Goat-anti-Mouse-IgG1-Cross-Adsorbed-Secondary-Antibody-Polyclonal/A-21127)
Secondary antibodies conjugated with Alexa-594 (https://www.thermofisher.cn/cn/zh/antibody/product/Donkey-anti-Goat-IgG-H-L-Cross-Adsorbed-Secondary-Antibody-Polyclonal/A-11058)

Eukaryotic cell lines

Policy information about cell lines

Cell line source(s)	HEK293T (ATCC® CRL-3216™), A549 (ATCC® CCL-185™), human embryonic stem cell line H1 (WiCell Research Institute), TRME hESCs cell line (modified from NKX2-5eGFP/w hESCs by Nan Cao's lab)
Authentication	Authentication of the cell lines was offered by the manufacturer or provider. The authentication included the viability, sterility, mycoplasma, surface marker testing, phenotyping by ICC or flow cytometry and molecular assays, and so on.
Mycoplasma contamination	Mycoplasma contamination was routinely monitored every week in our laboratory using Myco-Blue Mycoplasma Detector (Vazyme, D101). Cells were confirmed for the absence of mycoplasma contamination before every cellular experiments.
Commonly misidentified lines (See ICLAC register)	No commonly misidentified cell lines were used.

Flow Cytometry

Plots

Confirm that:

- [x] The axis labels state the marker and fluorochrome used (e.g. CD4-FITC).
- [x] The axis scales are clearly visible. Include numbers along axes only for bottom left plot of group (a 'group' is an analysis of identical markers).
- [x] All plots are contour plots with outliers or pseudocolor plots.
- [x] A numerical value for number of cells or percentage (with statistics) is provided.

Methodology

Sample preparation	Flow cytometry analysis was performed according to standard protocol of our previous studies. To detect membrane antigen, accutase-dissociated single cells were directly incubated with fluorochrome-conjugated antibody at 4°C for 1 hour. Then cells were washed three times, resuspended with ice-cold wash buffer. Isotype-matched normal IgG was used as negative control for all flow cytometry analysis experiments. For cell sorting assays, live cells were harvested, suspended in PBS containing 4% FBS and 1 μM thiazovivin, and stained as described above.
Instrument	Data was collected by a CytoFLEX S Flow Cytometer (Beckman Coulter). Cell sorting was performed by an FACS MoFlo Astrios EQs system (Beckman Coulter).
Software	Flow cytometric analysis was performed with FlowJo VX software.
Cell population abundance	In cell sorting experiments, CXCR4+ and CXCR4- cells comprised approximately ~6% and ~6% of the corresponding cell population , respectively, depending on the sample. An example is shown in Fig. 3b.

Gating strategy	Cells were gated on forward/side scatter parameters to exclude small debris and then gated on forward scatter height vs forward scatter area to exclude obvious doublet events.

☒ Tick this box to confirm that a figure exemplifying the gating strategy is provided in the Supplementary Information.
