# Supplementary material for: Parallel functional assessment of m6A sites in human endodermal differentiation with base editor screens
Source: Nat Commun. 2022 Jan 25;13:478. doi: 10.1038/s41467-022-28106-0 (PMC8789821; doi:10.1038/s41467-022-28106-0)
Supplement: Supplementary file 3 — Description of Additional Supplementary Files [file 41467_2022_28106_MOESM3_ESM.docx]

**Description of Additional Supplementary Files**

**Supplementary Data 1:** Information of the designed sgRNA library.

**Supplementary Data 2:** BE3 screening results in A549 cells.

**Supplementary Data 3:** Results of ABE screening in hESC cells.
